# Novel field-portable high-pressure adsorbent tube sampler prototype for the direct *in situ* preconcentration of trace compounds in gases at their working pressures: application to biomethane†

**DOI:** 10.1039/d2ra00601d

**Published:** 2022-03-30

**Authors:** Aurore Lecharlier, Hervé Carrier, Brice Bouyssiere, Guilhem Caumette, Pierre Chiquet, Isabelle Le Hécho

**Affiliations:** Universite de Pau et des Pays de l’Adour, E2S UPPA, CNRS, TOTAL, LFCR UMR 5150 BP 1155 Avenue de l'Université 64013 Pau Cedex France; Universite de Pau et des Pays de l’Adour, E2S UPPA, CNRS, IPREM UMR 5254 Technopôle Hélioparc, 2 Avenue du Président Angot 64053 Pau Cedex 09 France isabelle.lehecho@univ-pau.fr; Teréga 40 Avenue de l’Europe, CS 20 522 64010 Pau Cedex France

## Abstract

In Europe, renewable energy gases such as biomethane are aimed at substituting natural gas provided their stringent compliance to natural gas quality standards stipulating maximal levels of several chemical trace compounds (TC). Preconcentration is generally required to detect TC and inasmuch as biomethane is compressed for injection in the natural gas grid, preconcentration is commonly either done by collecting the bulk pressurized gas in a high-pressure cylinder or by first depressurizing it to collect a bulk volume in *e.g.* a gas sampling bag. Such whole gas samples are then transported to the lab and transferred to a preconcentration unit, entailing contamination and TC loss risks. Therefore, here a novel handy field-portable device for the direct *in situ* high-pressure preconcentration of TC is presented, enabling to sample gases at pressures up to 200 bar_a_ through a self-assembled Tenax®TA + Carbopack™X multibed adsorbent tube. The effect of the gas sampling pressure on the preconcentration of TC on adsorbent tubes was evaluated using a synthetic gas mixture containing 41 halogenated volatile organic compounds each at 1 ppm_mol_ in N_2_. At given normalized sampled volumes and in the pressure range 5–100 bar_a_ handled in French gas transport grids, the pressure had no influence on the preconcentration when the gas circulates through the adsorbent tubes and as long as the adsorbents are not saturated. Next, for the first time, a real biomethane stream was sampled using the novel direct high-pressure preconcentration method on Tenax®TA + Carbopack™X multibed adsorbent tubes, allowing to preconcentrate, in a single sampling run, a wide range of volatile organic TC. More than 26 distinct TC were detected, belonging to seven chemical families: alkenes, aromatics, alkanes (linear, cyclic and polycyclic), sulphur-compounds and terpenes, with linear alkanes (pentane, heptane, octane) and terpenes predominating. Semi-quantification indicated pentane, dimethylcyclopropane, hexane, heptane, octane, α-pinene and camphene are present at a ≤1 ppm_mol_ concentration threshold in the biomethane.

## Abstract 2

The circulating gas sampling pressure had no impact on the preconcentration of trace compounds on adsorbent tubes.

## Graphical abstract

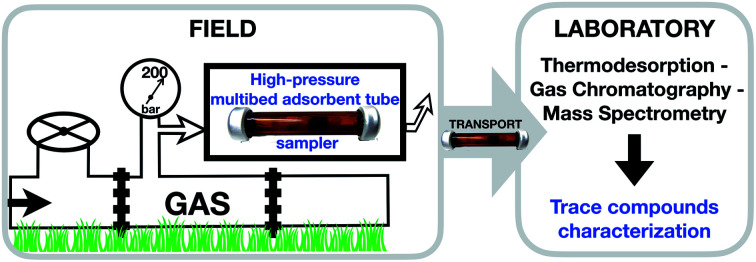

## 1. Introduction

In the present-day worldwide energy transition context, the share of renewable gases is meant to increase in the energy mix to tackle global greenhouse gases emissions. In France, the goal is to bring this share to 7 to 10% of the total gas consumption by 2030 depending on costs cuts.^1^ Biomethane and synthetic natural gas are pure methane (CH_4_) renewable gases with the same calorific value as fossil natural gas, produced in anthropogenically optimized processes from different biomass types, ideally from the wastes-sector, simultaneously contributing to the circular economy. Biomethane commonly refers to the purified methane fraction of biogas, a gas mixture composed of mainly <50% CO_2_ and >50% CH_4_ produced by the anaerobic digestion of humid organic matter by a microbially driven biochemical mechanism called methanization in controlled digesters^2,3^ or in landfills.^4,5^ Organic wastes (‘substrates’) used in anaerobic digesters include agricultural residues, manure, food-processing and catering wastes, organic and green municipal and household wastes, sewage sludge. Several technologies exist to upgrade biogas to biomethane by separation of the CO_2_ and CH_4_ fractions.^2,6–9^ Synthetic natural gas is obtained by pyrogasification and methanation of dry ligneous-cellulosic biomass (wood, straw, olive stones…).^10–13^

Those renewable methane streams are aimed at substituting or complementing natural gas in any of its applications (engines, boilers, cookers, fuels…). Stringent compliance of their quality to international natural gas quality standards is however required to guarantee their safe and sustainable injection in the natural gas transport grids^14^ or their use as vehicle fuels.^15^ Next to CH_4_ and depending on production conditions (digester or landfill, hydraulic retention time, temperature, humidity, pH…), substrates types, seasonal effects, and upgrading techniques, biomethane can contain low concentrations of various volatile compounds (trace compounds, TC) from diverse chemical families: alkanes, alkenes, terpenes, alcohols, aldehydes, ketones, ethers, esters, aromatics, halogenated organic compounds, organic and inorganic sulphur- and silicon-compounds^16–21^ and organic or inorganic metal and metalloid species.^22–24^ Observed concentrations range 30–35 000 μg m^−3^ (ref. 19) and <10–700 mg m^−3^ (ref. 20) for total volatile organic compounds; <100 μg_Si_ m^−3^ for total siloxanes^20^ and <300 μg_Si_ m^−3^ for total volatile methyl siloxanes;^17^ and 0.1–100 ng N m^−3^ for metallic trace compounds.^22^ Since natural gas grid quality standards stipulate maximal levels of among others ammonia, siloxanes, sulphur-, mercury- and halogenated-compounds to avoid those compounds inducing chemical reactions such as corrosion and abrasion that could damage gas infrastructures,^25^ sampling and quantifying biomethane's TC is crucial before grid injection. Odorant organic compounds of biomethane such as terpenes can also mask the odor of tetrahydrothiophene (THT) added to the gas for the safety of users (olfactive gas leak detection).^26^

Sampling, identification and quantification of biomethane's TC is difficult. The low concentrations not only imply high risks for TC loss by sorption to tubing, connectors and vessels in the sampling and analytical chains,^20,27–29^ but they often lie below the detection limits of analytical instruments, meaning a ‘preconcentration’ step is essential (the gas flows through a dedicated small-volume support with specific retention affinity for only given TC. Since the very volatile gas matrix itself (CH_4_) is not retained, TC are preconcentrated). Moreover, not any sampling nor preconcentration system is able to quantitatively trap all families of TC in one run in view of the complexity and diversity in physicochemical properties of the TC present (volatility, polarity, water solubility, reactivity…), resulting in different affinities and stabilities in the sampling entities.^20,21,27,29,30^ Lastly, monitoring TC in grid-quality compliant biomethane may imply the gas has already been compressed to the grid pressure (French distribution network: 4–6 bar_a_, transportation network: 8–80 bar_a_). To the authors' knowledge, biomethane has only been *in situ* sampled directly on the pipelines at the grid pressure (40 bar_a_) by Cachia *et al.*^22^ using a high-pressure acid bubbling impinger for the direct preconcentration of metallic TC in gas samples.^31^ So far, other reported determinations of TC in high-pressure gases (typically natural gas) have always been carried out by depressurization of the gas and preconcentration at atmospheric pressure: the gas is either depressurized *in situ* from the pipe after what the sampling system is installed at atmospheric pressure,^32^ or it is sampled at its grid pressure in surface-treated high-pressure stainless-steel cylinders subsequently transported to the lab for depressurization and preconcentration.^29,33–36^ Depressurization is detrimental to the preconcentration of TC since, assuming the ideal gas law *PV* = *nRT*, a dilution factor equal to the ratio of the high pressure to the pressure after depressurization leads to a concentration decrease of the TC, implying larger gas volumes have to sampled at atmospheric pressure than at high pressure to trap a given amount of TC. Next, a first whole gas sampling step in a high-pressure cylinder, cylinder transport to the lab, and then depressurization and transfer of the gas to the preconcentrating unit (*e.g.* sorbent tubes,^34^ cryogenic traps for metallic TC,^33^ amalgamation traps for mercury-TC^35,36^) has disadvantages. Firstly, transport of cylinders containing compressed flammable gas (CH_4_) must observe national regulations for the transport of dangerous goods. Secondly, transport entails a storage phase of the sample until analysis can be executed. Sorption losses of TC onto cylinders' inner surfaces or instabilities have been established for both metallic^34–36^ and non-metallic TC^21,29,30,37^ when complex gases such as natural gas or biomethane are stored in cylinders, despite appropriate surface polishing or passivation-treatments. Surface-treated cylinders are additionally expensive and the instability and cross-contamination of TC is worse in re-used than in brand new cylinders.^36^ Lastly, transfer of the gas from the cylinder to the preconcentration unit also increases the chances of sample loss or contamination due to leaks or sorption of TC on the gas transfer line materials. Having an easily field-implementable device at one's disposal that does not require solvents nor impingers, would avoid drawbacks diverted from the use of pressurized gas samples by enabling to sample target analytes at working pressures without depressurization; would simplify the sampling chain, avoid sample transfers and associated loss and contamination risks, avoid TC dilution by depressurization, diminish minimal sampling volumes and hence reduce sampling duration. To the authors' knowledge, such high-pressure preconcentration device does not exist.

Therefore, in this study, a novel handy field-portable sampling prototype for the direct *in situ* high-pressure preconcentration of non-metallic TC in gas samples at working pressures up to 200 bar_a_ is presented. To the authors' knowledge, this prototype is the first of its kind. Preconcentration takes place on self-developed multibed adsorbent tubes (MAT) packed with commercial adsorbents (Tenax®TA + Carbopack™X), placed in the high-pressure sampling prototype. The prototype was first validated by sampling a synthetic gas mixture containing 41 halogenated volatile organic compounds each at 1 ppm_mol_ in nitrogen through the MAT at pressures ranging 5–100 bar_a_. The effect of the gas pressure on the adsorption of the compounds was investigated to justify the use of the prototype. Next, biomethane was sampled in the prototype at a natural gas grid injection station at 40 bar_a_. Preconcentrated TC were characterized by thermal desorption of the adsorbent tubes hyphenated with gas chromatography and mass spectrometry. It was beyond the scope of this study to quantify TC identified and to determine TC's breakthrough volumes on adsorbent multibeds.

## 2. Materials and methods

### 2.1. Multibed adsorbent tubes

Multibed adsorbent tubes (MAT), whose theoretical working principle is explained in the ESI,† were self-assembled and conditioned as described in previous work.^38^ Briefly, empty amber glass tubes (ID 4.8 mm, L 44 mm, ActionEurope, Sausheim, France) are manually packed with commercial adsorbents from Supelco, Bellefonte, PA, USA (Table 1). The MAT held 14 ± 0.2 mg Tenax®TA (front bed) and 29 ± 0.2 mg Carbopack™X (back bed) and are further called ‘TA14-CpX29’. Each adsorbent is weighted and sucked up in the tube where it is secured between and separated from the other bed by untreated ∼4 mm long quartz wool plugs (Helios Italquartz™). To optimize the later thermal desorption of the MAT, adsorbent masses *m* were determined based on a fixed volume *V* = 0.05 cm^3^ for each bed and on the packing density *ρ* (Table 1): *m* = *ρV*. As such, each bed occupies a length of 3.4 ± 0.2 mm in the tube and it is ensured both lengths physically only occupy the central part of the tube that will be heated inside the thermodesorber. After packing, tubes are conditioned at 320 °C during 8.5 h under a continuous clean N_2_ flow as described earlier.^38^ The Tenax®TA conditioning temperature (320 °C) was used to condition the MAT as conditioning them at the higher Carbopack™X conditioning temperature (350 °C) would lead to irreversible thermal degradation of TA. As soon as the conditioning sequence is completed, tubes are sealed with aluminum crimp caps with PTFE/silicone/PTFE septa (11 mm, high temperature ultra-low-bleed silicone, ActionEurope) and stored until utilization in individual hermetic polyethylene zip bags in a larger zip bag in a desiccator at 4 °C as recommended by (ref. 27,39,40). Despite adsorbent tubes analyzed by thermodesorption can theoretically be re-used after quantitative thermodesorption and thermal reconditioning,^20,41,42^ here it was decided to only use new tubes for all sampling operations to avoid cross-contamination in the case thermodesorption of the initial sample was not quantitative and to avoid build-up of thermal degradation artefacts upon repeated conditioning cycles.

**Table 1:** Properties of commercial adsorbents used in the MAT

Adsorbent brand name	Nick-name	Matrix	Mesh size	Surface area (m^2^ g^−1^)	Packing density (g cm^−3^)	Conditioning *T* (°C)	Desorption *T* (°C)	Mass in the MAT (mg)	Position in the MAT
Tenax®TA	TA	Macroporous polymer (2,6-diphenyl-*p*-phenylene oxide)	60–80	35	0.28	320	300	14	Front bed
Carbopack™X	CpX	Graphitized carbon black	40–60	240	0.58	350	330	29	Back bed

### 2.2. High-pressure sampling prototype

**Fig. 1:**
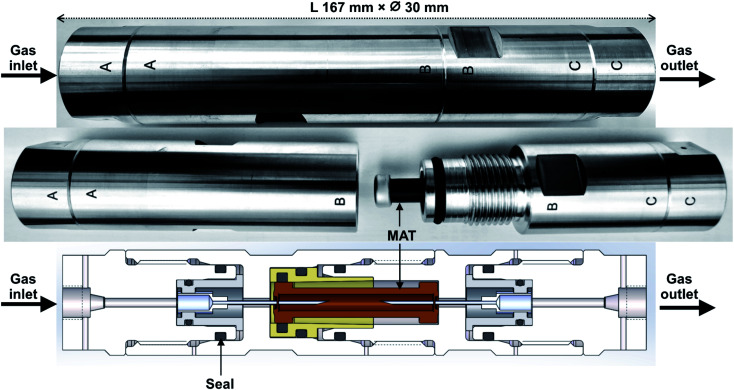
The high-pressure tube sampling prototype. Gas sampling direction is from A to C.

The novel field-portable high-pressure tube sampling prototype (HPTS) is derived from an existing patented device,^43^ both were manufactured by SANCHEZ TECHNOLOGIES (France). The HPTS is a purpose-built stainless-steel cylindrical envelope allowing to sample gas at pressures up to 200 bar_a_ through a self-assembled adsorbent tube using an equal-pressure gas flow design principle. The central parts AB and BC of the HPTS (Fig. 1) can be unscrewed to accommodate the adsorbent tube. Once the tube is placed, re-screwing parts AB and BC causes each of the fine beveled hollow needles located in both HPTS extremities (Fig. 1A and C), to pierce the inlet and outlet septa of the tube. Equal-pressure in- and outside the tube during sampling is achieved by a clever aperture in the upstream needle's base: when gas enters the HPTS at side A (Fig. 1), it not only flows throughout the needle and into the tube but also flows out of the needle's base into the space around the tube. The whole system is gas-tight. When the outlet HPTS valve is opened at side C (valves not shown on Fig. 1), high-pressure gas circulates through the tube and the total volume passed through can be controlled *via* a downstream flowmeter. The gas around the tube does not circulate. The HPTS itself has no flowrate limitations, those are set by the adsorbent tube adsorption and breakthrough properties.

### 2.3. Sampling

**Fig. 2:**
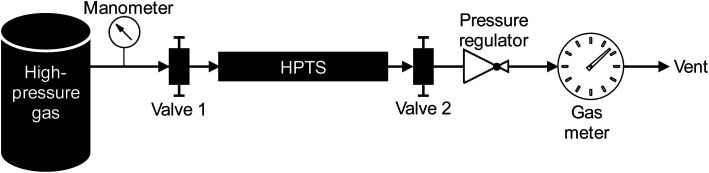
High-pressure preconcentration sampling chain.

The high-pressure preconcentration sampling chain is schematized in Fig. 2. The pressure of the high-pressure gas (either a synthetic gas cylinder for laboratory tests or a real gas during *in situ* sampling) is measured with a manometer (Leo2-Ei 0–300 bar_a_ Atex-certified, Keller, Switzerland) before the gas enters the MAT inside the HPTS. The MAT is oriented so that the gas first meets the front weak adsorbent bed (Tenax®TA). Downstream preconcentration, the gas is depressurized to atmospheric pressure (250–0 bar_a_ pressure regulator, Swagelok, France) before entering the drum-type gas meter (TG 0.5-polypropylene, Ritter, Germany). The gas meter is limited to flowrates of 1 L_N_ min^−1^ and the measure of the gas volume passed is independent on the gas composition. To sample a given gas volume, valves 1 and 2 (Fig. 2) are opened. Valve 1 is then closed to stop the sampling and the residual volume *V*_R_ trapped between valve 1 and the gas meter is also counted as contributing to the total sampling volume although once valve 1 is closed, the *V*_R_ circulates following a pressure gradient from the initial sampling pressure to atmospheric pressure. Henceforth, to sample a targeted gas volume *V*_T_, valve 1 has to be closed in advance when a volume *V* = *V*_T_ − *V*_R_ has passed through the gas meter. The gas volume effectively sampled under pressure is thus equal to *V*.

A bench supporting all sampling elements and tubing was built and is used in the lab as well as *in situ*. Connectors used are from Top Industrie (France) and Swagelok (France). Only stainless-steel tubing is used and attention is paid to always use clean tubing upstream the preconcentration in the HPTS. Before sampling a gas onto the MAT in the HPTS, the sampling chain without MAT is flushed with the gas to sample during few minutes to ‘accustom’ the sampling chain elements to the gas and to saturate potential TC-sorption sites on tubing upstream the HPTS. Between subsequent sampling operations of gases of different composition, the HPTS is flushed with pure nitrogen (99.999% purity) during ≥60 min to remove residual sample traces and avoid sample cross-contamination. All sampling operations are performed at ambient temperature. All lab- and field-sampled adsorbent tubes were stored in individual hermetic polyethylene zip bags in a larger zip bag in a desiccator at 4 °C until analysis and were analyzed within 36 hours as recommended by (ref. 27,39,40).

#### 2.3.1. Synthetic gas sampling

The HPTS containing a TA14-CpX29 MAT was first tested in the lab using a pressurized certified synthetic gas mixture (SGM) containing 41 halogenated volatile organic compounds (HVOC) each at 1 ppm_mol_ in nitrogen (Table 2) (‘TO-14A 41 Component Mix’, Scott Airgas Specialty Gases, Plumsteadville, USA, purchased from Restek, France). Yet the HPTS withstands up to 200 bar_a_, high-pressure sampling tests were only performed up to 100 bar_a_ at different test-pressures (Table 3) covering the pressure range used in the French gas distribution grids (4–6 bar_a_) and transport grids (8–80 bar_a_). A high-pressure regulator was connected to the SGM cylinder to achieve the desired test-pressure which was also controlled by a digital pressure sensor (accuracy ±0.05 bar_a_) (Scaime).

**Table 2:** The 41 HVOC present in the SGM used, listed in order of increasing boiling points. Note 1,2-dichloropropane was never detected on the TA14-CpX29 MAT despite both adsorbents should enable fair adsorption and recovery (>80%) of this compound.^44^

Compound	Boiling point (°C, at P_atm_)	Molecular mass (g mol^−1^)
Dichlorodifluoromethane	−30.0	120.9
Chloromethane	−23.8	50.5
Chloroethene	−13.4	62.5
1,3-Butadiene	−4.4	54.1
1,2-Dichloro-1,1,2,2-tetrafluoroethane	3.6	170.9
Bromomethane	4.0	94.9
Chloroethane	12.5	64.5
Trichlorofluoromethane	23.8	137.4
1,1-Dichloroethene	32.0	96.9
Dichloromethane	39.6	84.9
1,1,2-Trichloro-1,2,2-trifluoroethane	48.0	187.4
1,1-Dichloroethane	57.0	99.0
*cis*-1,2-Dichloroethene	60.2	96.9
Trichloromethane	61.2	119.4
1,1,1-Trichloroethane	74.0	133.4
Tetrachloromethane	76.7	153.8
Acrylonitrile	77.0	53.1
Benzene	80.0	78.1
1,2-Dichloroethane	84.0	99.0
Trichloroethene	87.2	131.4
1,2-Dichloropropane (*absent*)	96.0	113.0
*cis*-1,3-Dichloropropene	104.0	111.0
Toluene	111.0	92.1
*trans*-1,3-Dichloropropene	112.0	111.0
1,1,2-Trichloroethane	112.5	133.4
Tetrachloroethene	121.1	165.8
Chlorobenzene	131.0	112.6
1,2-Dibromoethane	131.5	187.9
Ethylbenzene	136.0	106.2
*p*-Xylene	138.0	106.2
*m*-Xylene	139.0	106.2
*o*-Xylene	144.0	106.2
Styrene	145.0	104.2
1,1,2,2-Tetrachloroethane	146.0	167.8
1,3,5-Trimethylbenzene	164.7	120.2
1,2,4-Trimethylbenzene	170.0	120.2
1,3-Dichlorobenzene	172.0	147.0
1,4-Dichlorobenzene	174.0	147.0
1,2-Dichlorobenzene	180.2	147.0
1,2,4-Trichlorobenzene	213.5	181.4
Hexachloro-1,3-butadiene	215.0	260.8

**Table 3:** Experimental conditions for the HPTS lab validation. *n* = amount of successful replicates

Test-condition	Test-pressure (±0.05 bar_a_)	Theoretical sampled volume (L_N_)	Average effective sampled volume (L_N_)	Standard deviation effective sampled volume (L_N_)
A	5	2	2.01 (*n* = 3)	0.02
	40		2.06 (*n* = 2)	0.02
	100		2.22 (*n* = 3)	0.57
				
B	5	5	5.00 (*n* = 4)	0.02
	40^a^		4.87 (*n* = 3)	0.05
	68		4.80 (*n* = 1)	—
	74		5.04 (*n* = 1)	—
				
C	40	1	0.98 (*n* = 2)	0.06
		2	2.06 (*n* = 2)	0.02
		5	5.02 (*n* = 1)	—
				
D	5	2	2.01 (*n* = 3)	0.02
		5	5.00 (*n* = 4)	0.02
		6	6.01 (*n* = 3)	0.02

a On the *n* = 3 replicates, two were performed at 40 bar_a_ and one at 39 bar_a_.

The effect of the circulating gas pressure on the preconcentration (adsorption) of the 41 HVOC on the MAT was investigated by sampling given gas volumes (2 and 5 L_N_) at different test-pressures at a flowrate of 1 L_N_ min^−1^ through the MAT. To ensure these pressure-effect tests were performed in conditions of non-saturation of the adsorbents in the MAT, different volumes were also sampled at given pressures (5 and 40 bar_a_) at 1 L_N_ min^−1^ to verify the saturation point of the breakthrough curve was not reached for the 41 HVOC (Table 3). Sampling operations were all executed at constant ambient temperature (20 °C).

#### 2.3.2. *In situ* biomethane sampling

Next, the HPTS was used *in situ* to preconcentrate TC in a biomethane injected at 40 bar_a_ in the French natural gas transport grid. The biomethane sampled is produced by biogas upgrading at an anaerobic digestion plant gathering agricultural, manure (duck, cow, sheep) and food processing residues. Biogas is upgraded by water washing in a fluidized bed (scrubber). Water streams downwards while biogas streams upwards. Water-soluble CO_2_ and H_2_S gas components dissolve in water while CH_4_ does not and moves to the top of the scrubber where it is evacuated towards the natural gas grid injection pool and dried *via* pressure swing adsorption on regenerable hydrophilic silica beads.

The HPTS containing a TA14-CpX29 MAT was connected to the biomethane grid injection pipe at 40 bar_a_ using a clean 2.5 m long stainless-steel tube dedicated to this site. The sampling point was located upstream the THT odorization point. 2 L_N_ were collected through the HPTS directly at 40 bar_a_ on 6 MAT replicates at 1 L_N_ min^−1^. Six other MAT replicates were sampled after depressurization at 1.45 bar_a_ with 2 L_N_ at 1 L_N_ min^−1^ from the same sampling point. All samples were taken the same day within 4 hours at ambient outdoor temperature (8.2 ± 0.1 °C). Before and after sampling, adsorbent tubes were transported from and to the lab in individual hermetic polyethylene zip bags in a larger zip bag in a polystyrene box filled with carbon dioxide dry ice.

### 2.4. Analysis

All sampled MAT are analyzed *via* TD-GC-MS: thermal desorption (nCx Instrumentation, Garlin, France, ‘nCx-TD’ thermodesorber prototype) coupled to gas chromatography (Agilent 6890A GC) and mass spectrometry detection with quadrupole mass filter (Agilent 5973Network Mass Selective Detector) programmed as in Table 4 using the MSD ChemStation E.02.02.1431 software (Agilent) and the NIST Mass Spectral Search Program version 2.0 d, 2005. Each MAT is placed in the thermodesorber in the reverse direction as compared to the gas sampling direction. The nCx-TD prototype was presented in previous work^38^ and the chromatographic peak resolutions, limits of detection and repeatabilities obtained with this TD-GC-MS analytical chain have also been presented in (ref. 38). Note the 200 °C temperature programmed in the nCx-TD corresponds to an effective desorption temperature of 300 °C inside adsorbent tubes. The MAT desorption temperature is 300 °C since desorbing MAT at 330 °C (desorption temperature of CpX, Table 1) would induce thermal degradation of TA (desorption temperature 300 °C) with associated injection of its thermal degradation products in the GC-MS and falsification of the analytical results as well as irreversible TA damage.

**Table 4:** TD-GC-MS instrument parameters

Instrument	Parameter	Value/reference
nCx-TD prototype nCx instrumentation	Safe temperature	35 °C
	Temperature	200 °C
	Stabilization time	15 s
	Pressure	1170 mbar
	Injection time	10 s
GC Agilent 6890A	Inlet temperature	230 °C
	Inlet septum	Premium inlet septa, bleed/temp optimized, non-stick (Agilent)
	Inlet liner	Ultra inert liner, splitless, single taper, no wool, 4 mm ID (Agilent)
	Split ratio	1 1
	Split flow	1.5 mL min^−1^
	Carrier gas	Helium (quality detector 5.0, linde, France)
	Gas saver	Off
	Column	HP-5MS, 30 m × 250 μm ID × 0.25 μm film thickness (Agilent)
	Constant flow in column	1.5 mL min^−1^
	Carrier gas linear velocity in column	44 cm s^−1^
	Oven	30 °C (4 min) – 10 °C min^−1^ – 250 °C (5 min)
MS Agilent 5973Network Mass Selective Detector	Source temperature	230 °C
	Quadrupole temperature	150 °C
	GC-MS interface temperature	280 °C
	Electron impact mode	70 eV
	Electron multiplier voltage	Relative voltage (106 = 1871 V)
	Acquisition mode	Scan
	Scan range	10–450a.m.u.
	Sampling rate	3.28 scan s^−1^
	Threshold	100 counts

### 2.5. Calculations

In real biomethane samples, the relative abundance (RA, %) of each TC *i* (*i* = {1 → *n*}) identified upon TD-GC-MS analysis of the sampled MAT, was calculated as follows:
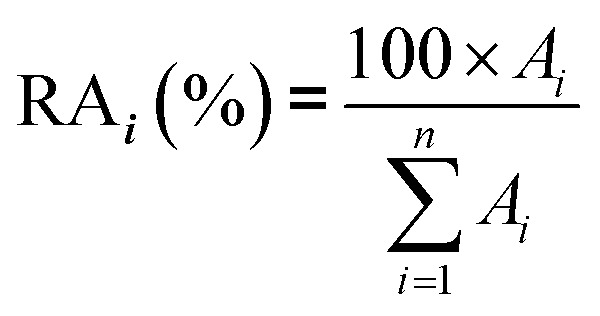
with *A*_*i*_ the average chromatographic peak area of compound *i* across all replicates on the total ion current chromatograms (TIC). For the per-chemical family RA (*e.g.* alkanes), *n* = the number of alkanes found in the sample. For the global RA in the whole sample, *n* = the total number of TC identified.

## 3. Results and discussion

### 3.1. High-pressure sampling prototype validation

The equal-pressure working principle of the novel HPTS allowed to sample pressurized gases through MAT without any physical damage to the glass tubes: tubes do not break and adsorbent beds do not move inside their tubes. The HPTS is handy and field-portable, allowing easy sampling at any gas production site at any pressure up to 200 bar_a_. Note the sampling chain in Fig. 2 is currently equipped to work at 200 bar_a_ yet it can easily be adapted to work at higher pressures up to 1000 bar_a_.

### 3.2. Multibed adsorbent tubes adequacy

In this study, the SGM used was chosen for its 41 HVOC trace compounds (Table 2), some of which may be present in real biomethane samples.^16,21,45^ The TA14-CpX29 MAT configuration proved suitable to adsorb and desorb all HVOC present in the SGM at all test-pressures in the range 5–100 bar_a_ with the exception of chloromethane which was never detected (Table SI-1 in the ESI†). Tenax®TA and Carbopack™X are indeed both too weak to adsorb and recover the highly volatile and small chloromethane molecule (recovery < 20%^44^). Stronger adsorbents than Carbopack™X could be used as back bed in MAT when targeting very volatile and small compounds such as chloromethane. Care should nevertheless be taken that such stronger adsorbents also enable recovery of the compounds upon analysis.

**Fig. 3:**
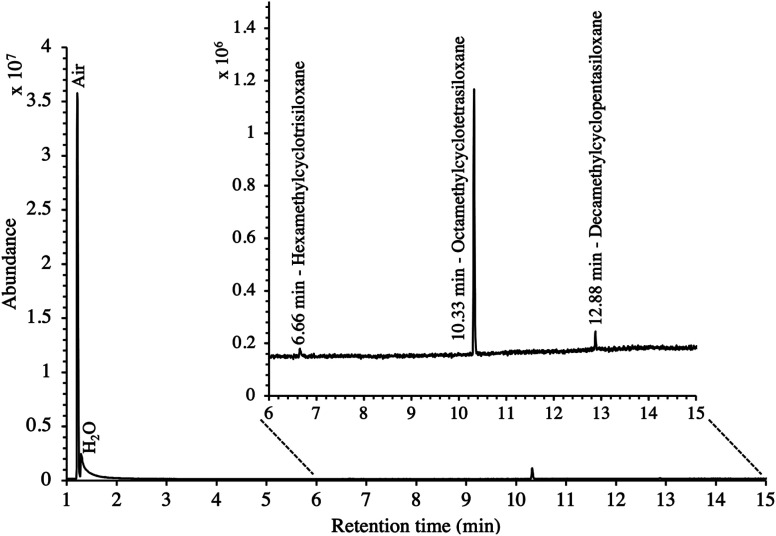
TIC of a new blank TA14-CpX29 MAT with indication of septum-released siloxane background contaminants.

New blank TA14-CpX29 MAT were also TD-GC-MS analyzed and were free of any inherent contaminant with the exception of siloxanes released from the PTFE/silicone/PTFE septa used to crimp-cap the tubes (Fig. 3), indicating the tube assembly and conditioning procedure was adequate. Notwithstanding, other septa materials should be considered to achieve zero-release of impurities from tube materials while still offering softness and gas-tightness after needle piercing.

### 3.3. Influence of the gas pressure on the preconcentration

**Fig. 4:**
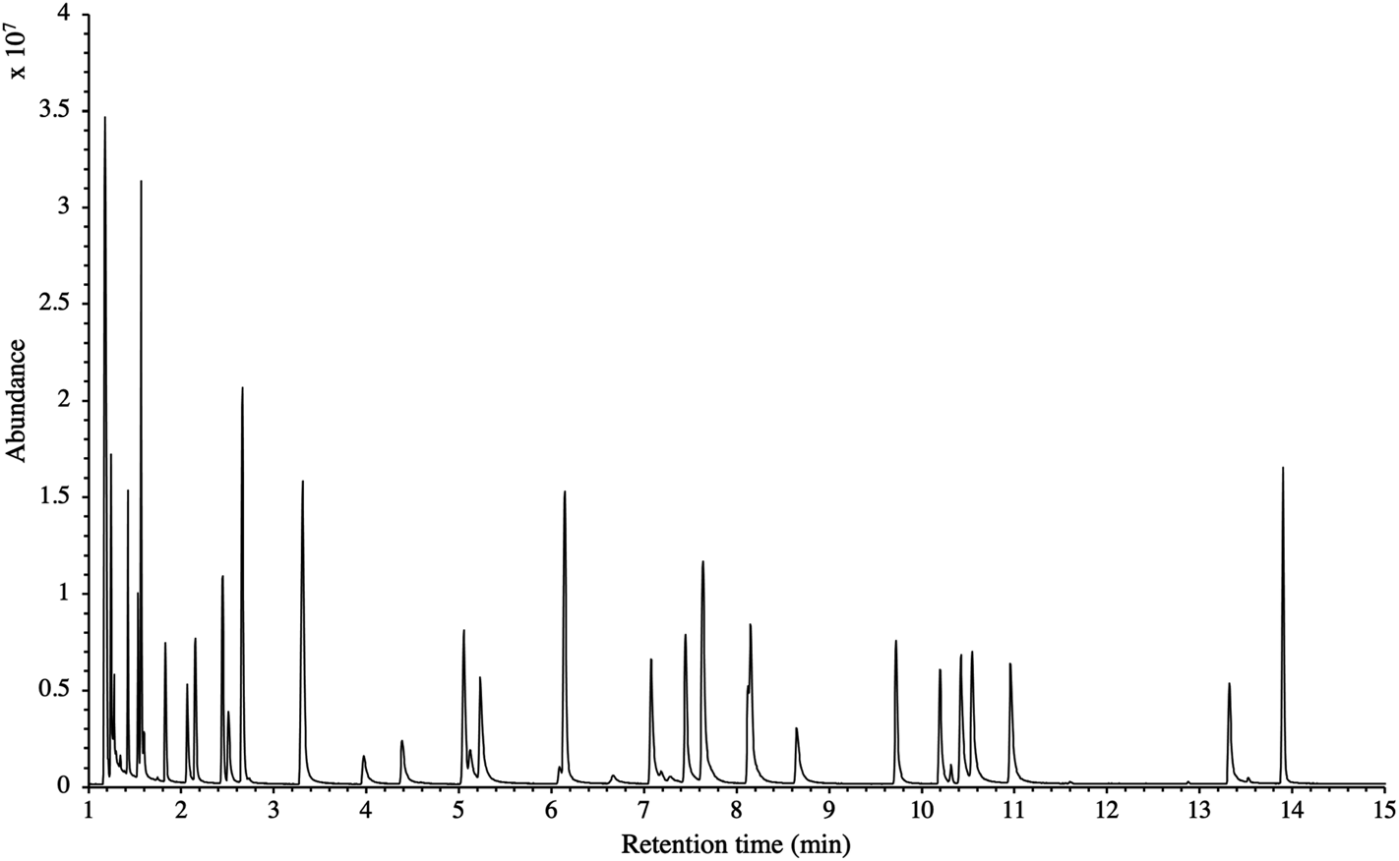
TIC of the 41 HVOC SGM sampled (2 L_N_) at 100 bar_a_ on TA14-CpX29 MAT in the HPTS. Retention times are given in Table SI-1†.

**Fig. 5:**
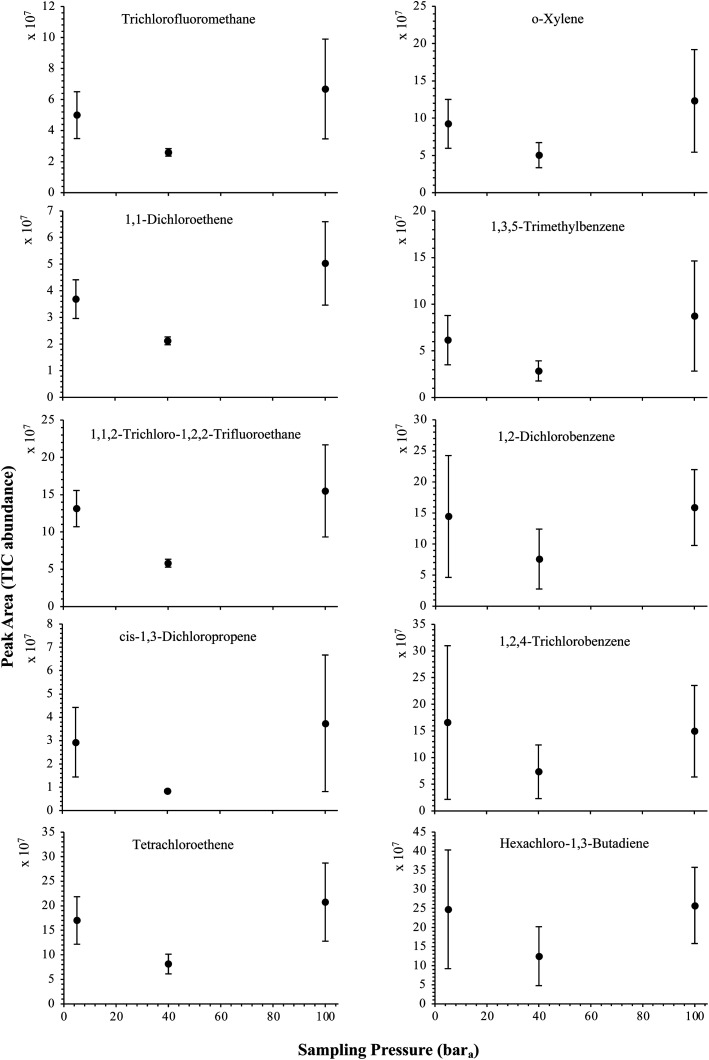
High-pressure adsorption isotherms of 10 randomly selected HVOC (out of the 41) for test-condition A (2 L_N_ of the SGM sampled at 5, 40 and 100 bar_a_ on TA14-CpX29 MAT). Average peak area with indication of the standard deviation. The remaining HVOC are plotted in the ESI : Fig. SI-1†.

**Fig. 6:**
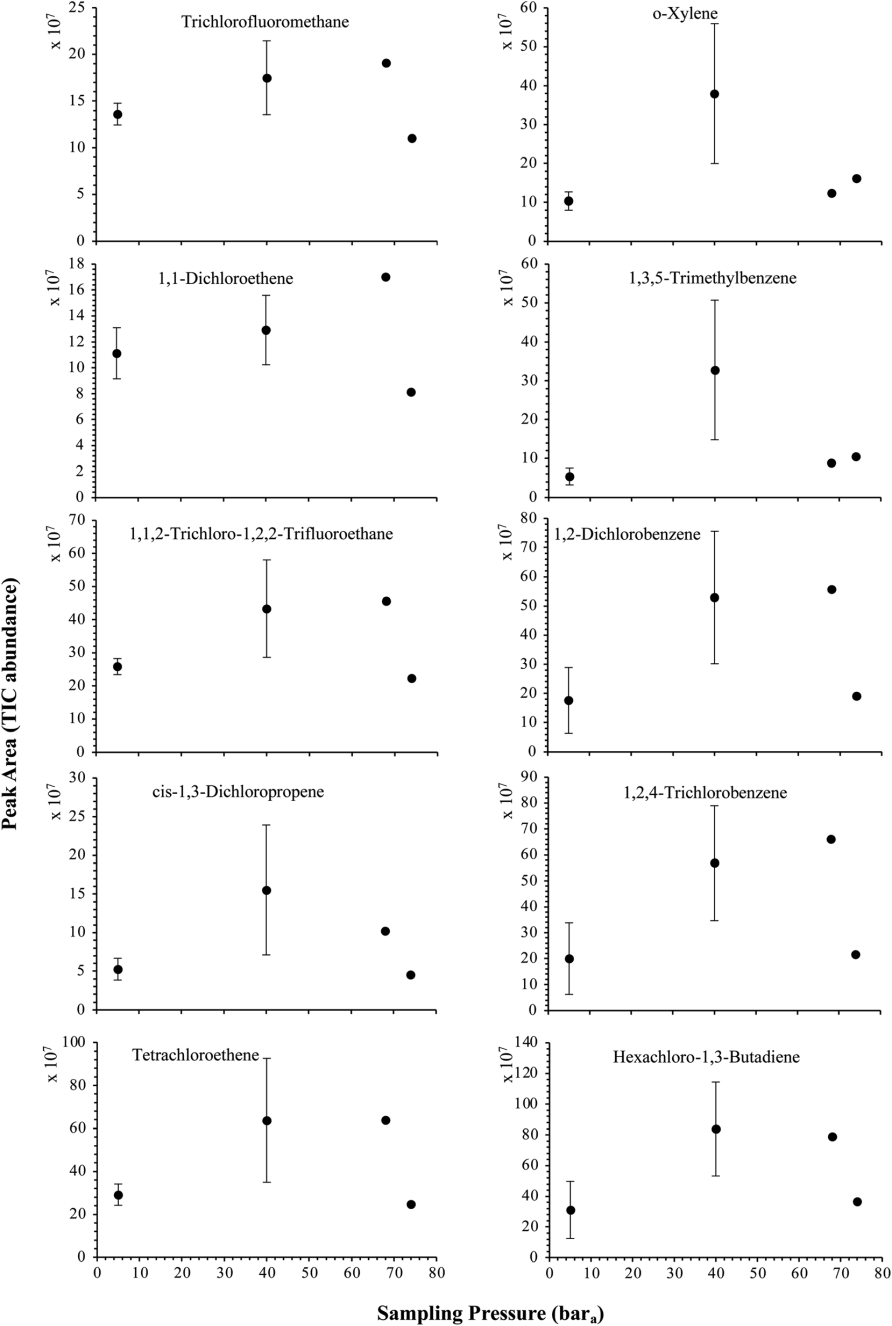
High-pressure adsorption isotherms of 10 randomly selected HVOC (out of the 41) for test-condition B (5 L_N_ of the SGM sampled at 5, 40, 68 and 74 bar_a_ on TA14-CpX29 MAT). Average peak area with indication of the standard deviation. The remaining HVOC are plotted in Fig. SI-2†.

**Fig. 7:**
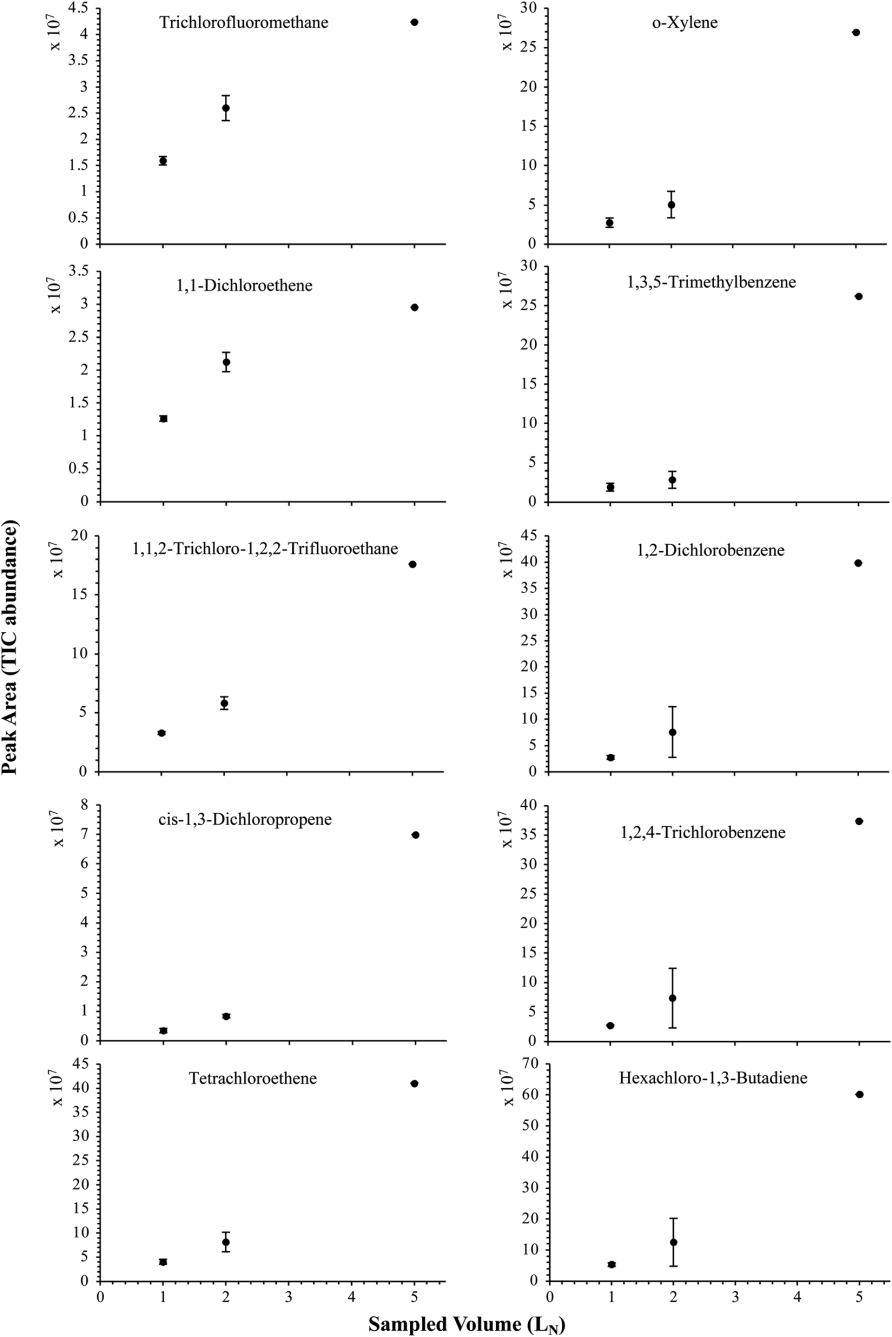
Partial breakthrough curves for 10 randomly selected HVOC (out of the 41) for test-condition C (1, 2 and 5 L_N_ of the SGM sampled at 40 bar_a_ on TA14-CpX29 MAT). Average peak area with indication of the standard deviation. The remaining HVOC are plotted in Fig. SI-3†.

**Fig. 8:**
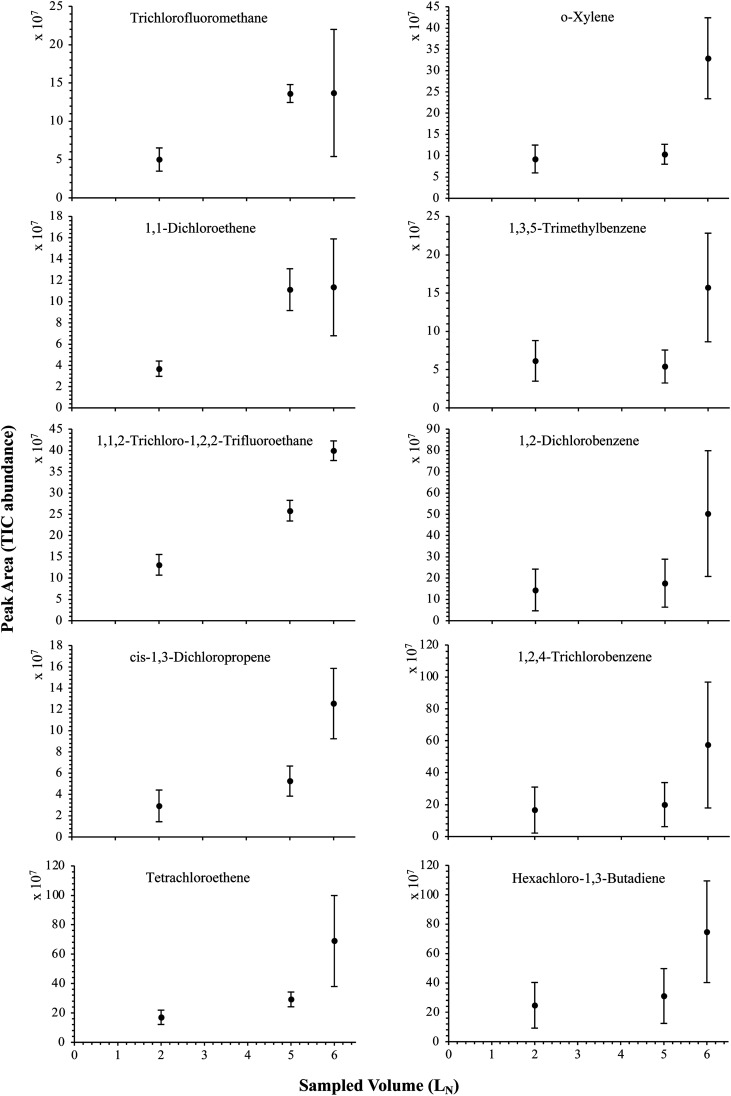
Partial breakthrough curves for 10 randomly selected HVOC (out of the 41) for test-condition D (2, 5 and 6 L_N_ of the SGM sampled at 5 bar_a_ on TA14-CpX29 MAT). Average peak area with indication of the standard deviation. The remaining HVOC are plotted in Fig. SI-4†.

The influence of the gas pressure on the preconcentration of the 41 HVOC of the SGM on the adsorbent materials upon circulating the gas through the HPTS was investigated. In Fig. 4, the total ion current chromatogram (TIC) obtained from the TD-GC-MS analysis of a TA14-CpX29 MAT sampled with 2 L_N_ of the 41 HVOC SGM at 100 bar_a_ is depicted. From the TIC resulting from each high-pressure test-condition listed in Table 3, the chromatographic peak areas were recorded for each HVOC. In Fig. 5 and 6, the average chromatographic peak areas for the replicates at test-conditions A and B respectively, have been plotted for each HVOC against the sampling pressure of the SGM on the TA14-CpX29 MAT. Results in Fig. 5 and 6 present relatively high standard deviations due to the poor nCx-TD repeatability which was demonstrated in previous work.^38^ In view of the systematic overlap of peak area-error bars (standard deviations) between the different test-pressures in Fig. 5 (2 L_N_ sampled at different pressures) and Fig. 6 (5 L_N_ sampled at different pressures), no effect of the gas sampling pressure on the preconcentration of the 41 HVOC on the TA14-CpX29 MAT could be established between 5 and 100 bar_a_ when the gas circulates through the MAT and as long as the MAT are not saturated. Results also demonstrated the gas sampling pressure had no effect on the chromatographic retention time of the HVOC (Table SI-1†). The non-saturation of the MAT by the trace HVOC studied was evaluated by test-conditions C and D (Table 3) where growing SGM volumes were sampled on the MAT at two given pressures: 1, 2 and 5 L_N_ at 40 bar_a_ (Fig. 7) and 2, 5 and 6 L_N_ at 5 bar_a_ (Fig. 8) respectively. For each HVOC studied, Fig. 7 and 8 plot the average chromatographic peak areas for the replicates at test-conditions C and D respectively, against the sampled volume of the SGM on the MAT. While the preliminary shape of a breakthrough curve, or adsorption isotherm, appears for each HVOC in Fig. 7 and 8, it is not possible to identify the isotherm type each compound follows with regards to *e.g.* the IUPAC adsorption isotherm classification^46^ since too few measurement points were obtained to draw a complete isotherm. Nevertheless, it can be claimed that sampling 2 or 5 L_N_ of the 41 HVOC SGM does not lead to saturation of the sorption sites on the TA14-CpX29 MAT as the pseudo-isotherms in Fig. 7 and 8 do not reach a plateau at those volumes for all HVOC studied. For the most volatile HVOC (from dichlorodifluoromethane to 1,1-dichloroethane in Table 2), saturation may start at 6 L_N_ (Fig. 8). Dissimilarities in adsorption behavior and adsorbent surface coverage mechanisms between the 41 HVOC studied on the TA14-CpX29 MAT, are suggested by the potentially different adsorption isotherms in Fig. 7 and 8, although investigating those differences goes beyond the scope of this study.

To the authors' knowledge, the pressure effect studied here has not been previously investigated. Thermodynamic researches on high-pressure adsorption of gases on microporous adsorbents have mainly focused on gases like N_2_, CO_2_, CO, CH_4_, Ar and H_2_ for industrial gas separation or enhanced gas storage purposes.^47–49^ Few publications^50^ have dealt with other gaseous species such as the 41 HVOC studied here. Furthermore, closed {gas (adsorbate) – adsorbent} systems in equilibrium conditions and at above-critical temperatures are generally assumed. The high-pressure preconcentration system considered in the present study is fundamentally different inasmuch as the gas circulates through an adsorbent tube at the same pressure as the pressure surrounding it, under non-equilibrium and non-saturation conditions at ambient temperatures and since adsorbates are not the bulk N_2_ nor CH_4_ matrix but the 41 HVOC. The absence of pressure effect on adsorption observed here therefore contrasts with the established conclusions from high-pressure adsorption thermodynamics where adsorption of TC tends to increase with the gas pressure.^47–50^ The observed absence of pressure effect may be due to several factors. Firstly, the test-pressure range of 5–100 bar_a_ handled here may possibly be too narrow to reveal any pressure effect. Nonetheless, this pressure range was chosen to represent pressures used in the French gas distribution and transport grid, thus for this application, testing higher pressures may be irrelevant. Secondly, it is questionable whether the pressure could exert a prejudicial influence on the porous structure of the adsorbents in the MAT, such as modifying the specific surface area or the specific pore volume. This last assumption is however unlikely since Salem *et al.*^47^ studied high-pressure induced changes in pore size distribution and in structure of microporous adsorbents (active carbon and zeolite 13X) and found high-pressure adsorption did not modify the porous structure of the microporous adsorbents.

The results presented here therefore suggest an efficient and non-selective preconcentration of TC from gaseous samples on MAT in the HPTS independently from the pressure of the circulating gas since all HVOC studied were equally and proportionately trapped on the MAT at all test-pressures. This high-pressure preconcentration sampling method is hence justified and does not need particular preliminary pressure-dependent calibration operations as long as the gas circulates through the MAT and that the total sampled volume does not saturate the adsorbents.

### 3.4. HPTS-application to biomethane's trace compounds characterization

TC in the biomethane sampled directly *in situ* at 40 bar_a_ or after depressurization at 1.45 bar_a_ on TA14-CpX29 MAT in the HPTS were characterized by TD-GC-MS of the sampled MAT. The goal was to qualitatively screen a large spectrum of TC-families rather than to focus on a single family or a single TC (multibed principle).

**Fig. 9:**
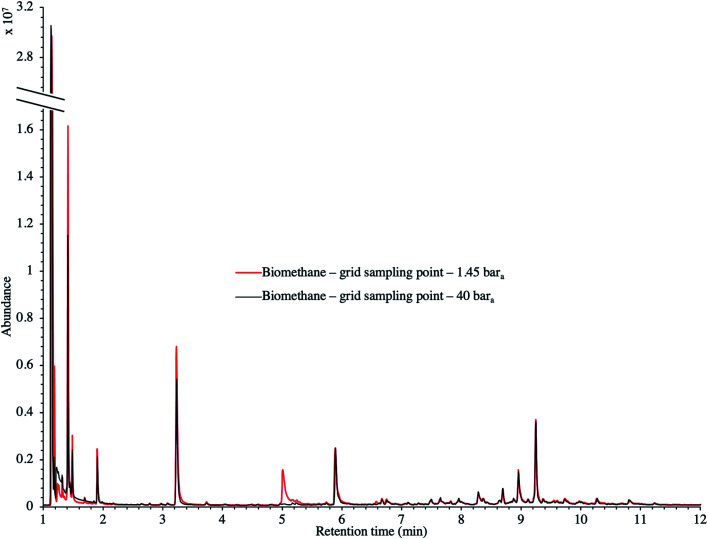
TIC of two biomethane samples: 2 L_N_ collected on TA14-CpX29 MAT at 1 L_N_ min^−1^ at 1.45 bar_a_ after depressurization *versus* directly at 40 bar_a_ in the HPTS.

**Fig. 10:**
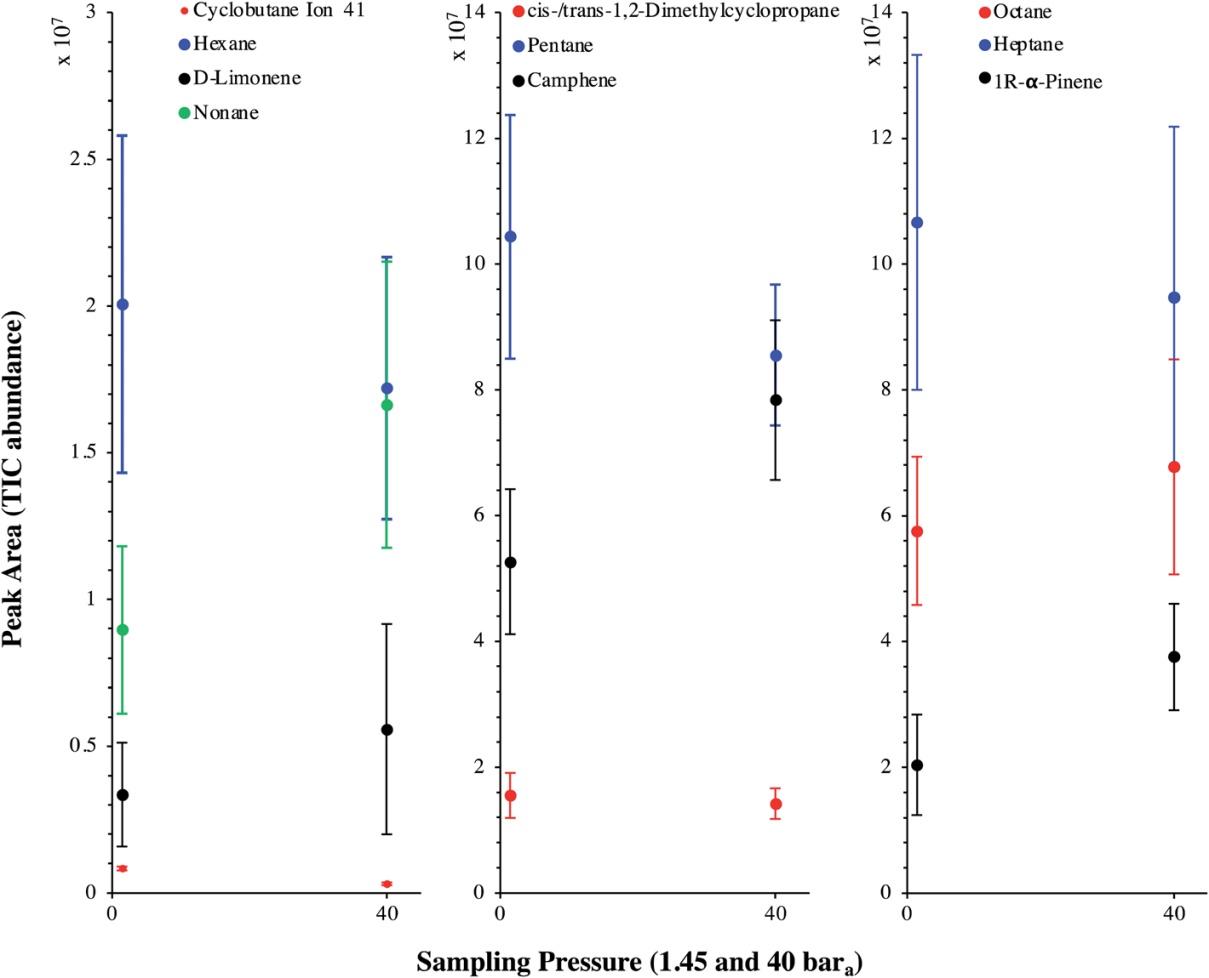
Average chromatographic peak area, with indication of the standard deviation, of 10 TC identified in the TIC of both biomethane sample types: 2 L_N_ collected on TA14-CpX29 MAT at 1 L_N_ min^−1^ at 1.45 bar_a_ after depressurization (*n* = 5 successful replicates) *versus* directly at 40 bar_a_ in the HPTS (*n* = 6 successful replicates).

Fig. 9 presents the TIC recorded for one biomethane sample replicate preconcentrated directly at 40 bar_a_*versus* a replicate preconcentrated after depressurization at 1.45 bar_a_. Disregarding the toluene peak at 5.03 min being large in the sample preconcentrated after depressurization, the visual evaluation of Fig. 9 suggests no striking difference in TIC signal intensities between the two samples, confirming the aforementioned statement (Section 3.3) that the sampling pressure has *a priori* no significant effect on the preconcentration of TC in gas samples under the sampling conditions handled here (gas circulates through unsaturated adsorbents). The relatively large toluene peak in the sample taken at 1.45 bar_a_ was confirmed to stem from a toluene-contamination of the tubing and connectors of the depressurization bench (results not shown). This highlights the critical advantage of sampling a compressed gas as close as possible to its source when targeting TC, *i.e.* at its grid pressure to shorten the sampling chain and avoid contamination risks in surplus equipment. Impressions from Fig. 9 are corroborated by Fig. 10 where the average chromatographic peak area of 10 TC identified in all biomethane replicates preconcentrated directly at 40 bar_a_*versus* at 1.45 bar_a_, are plotted against the sampling pressure. Again, the overlap of standard deviation error bars and the sometimes increasing – sometimes decreasing peak area trend in Fig. 10 do not allow to authenticate a significant effect of the sampling pressure on the preconcentration.

**Fig. 11:**
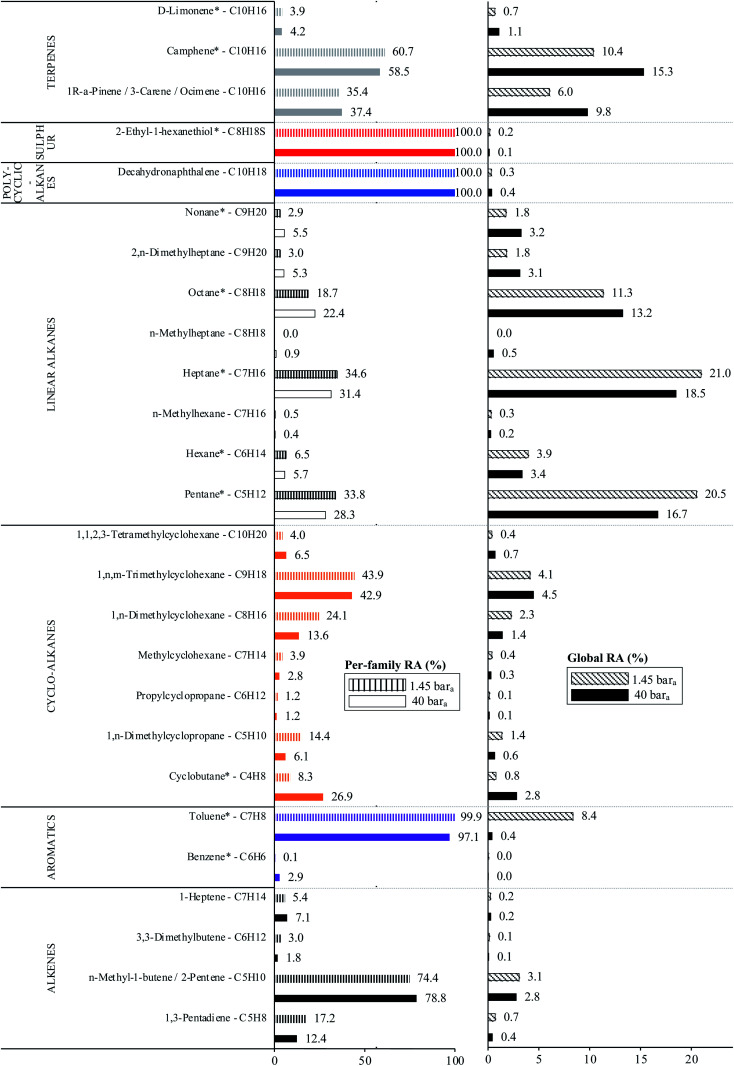
Per-chemical family and global relative abundances (RA, %) of molecular formulas, with indication of the potential corresponding TC, identified in both biomethane sample types: 2 L_N_ collected on TA14-CpX29 MAT at 1 L_N_ min^−1^ at 1.45 bar_a_ after depressurization (*n* = 5 successful replicates) *versus* directly at 40 bar_a_ in the HPTS (*n* = 6 successful replicates). Compounds marked with a “*” are unequivocally identified.

The average TC's biomethane composition was determined for the samples preconcentrated at 40 *versus* at 1.45 bar_a_ from the peaks identified in the respective replicates (Fig. 11). Importantly, the HP-5MS chromatographic capillary column used was chosen for its non-polar stationary phase and associated ‘universal’ retention properties enabling to analyze a wide range of compounds in a broad polarity and volatility range such as found in biomethane samples. Disadvantageous to this column was nevertheless the co-elution of several TC and the difficult unambiguous peak identification with the NIST database. Therefore, for clarity and to avoid misidentification, molecular formulas are given in Fig. 11 to represent the TC determined. An unequivocal compound identification could be done for those labeled with a “*” on Fig. 11: benzene, toluene, cyclobutane, pentane, hexane, heptane, octane, nonane, 2-ethyl-1-hexanethiol, camphene, d-limonene. For the other TC whose identification was equivocal between various compounds having the same molecular formula but different structural formulas, the main corresponding compound has been labeled on Fig. 11 as an indication. The per-family and global relative abundance (RA) of each TC (or each molecular formula) are given in Fig. 11 (chemical families include alkenes, aromatics, cyclo-alkanes, linear alkanes, polycyclic alkanes, sulphur-compounds, terpenes). For molecular formulas with several occurrences (chromatographic peaks), the average chromatographic peak areas of all occurrences were summed up (‘*A*_i,sum_’) and the corresponding RA was calculated as 
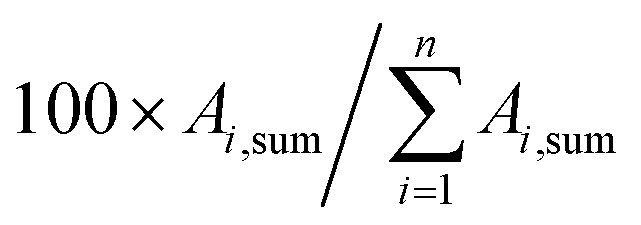
. Importantly, the RA are only given in Fig. 11 as a rough guide to decipher notable trends in dominant TC present in the biomethane since so far, no TC quantification was done owing to a lack of time in this research project. RA's are nowise proportional to TC's concentrations in view of the differences in ionization efficiency between the TC in the mass spectrometer detector yielding signal intensity-differences in the TIC even at equal concentration.

In the biomethane sampled, at least 26 distinct TC were found to belong to seven chemical families: alkenes, aromatics, cyclo-alkanes, linear alkanes, polycyclic alkanes, sulphur-compounds, terpenes (Fig. 11). No qualitative composition difference was noticed between the biomethane preconcentrated directly at 40 bar_a_ and the one preconcentrated after depressurization at 1.45 bar_a_ with the exception of some C_8_H_18_ linear alkanes absent from the samples taken at 1.45 bar_a_. Their absence may be due to sorption losses on tubing and connectors of the depressurization bench, once again underlining the importance of shortening the sampling chain upstream preconcentration. Among alkenes, C_5_H_10_ compounds were dominant. Among aromatics, solely benzene and toluene traces were found, with toluene reaching higher levels (recall the toluene contamination in the sample taken after depressurization). The cyclo-alkanes diversity was the highest with 7 distinct molecular formulas identified from C_4_H_8_ to C_10_H_20_. C_9_H_18_ species were the dominant cyclo-alkanes. Linear alkanes were also diversified with pentane, hexane, heptane, octane and nonane and several other C_7_H_16_, C_8_H_18_ and C_9_H_20_ species. Pentane and heptane were the most abundant linear alkanes. Polycyclic alkanes only counted a C_10_H_18_ species, and a single sulphur-compound was also identified (2-ethyl-1-hexanethiol). Finally, at least 5 terpenes (C_10_H_16_) were detected: camphene (the most abundant), d-limonene, α-pinene, 3-carene and ocimene. Regarding global relative abundances (Fig. 11), and momentarily overlooking the differences in ionization efficiency between the TC, linear alkanes (pentane, heptane, octane) and terpenes seem to be the predominant TC in the biomethane. Those two families are often reported as abundant in biogases and biomethane,^16,45^ terpenes being known to typically originate from vegetal matter^26,45^ which may enter the anaerobic digester considered in this study through the agricultural crop and food processing residues. No silicon-containing compounds were found in this biomethane, agreeing with other studies on farm- or agricultural-sourced biogas where silicon-compounds are generally absent or present at lower concentrations than other TC.^18^

**Fig. 12:**
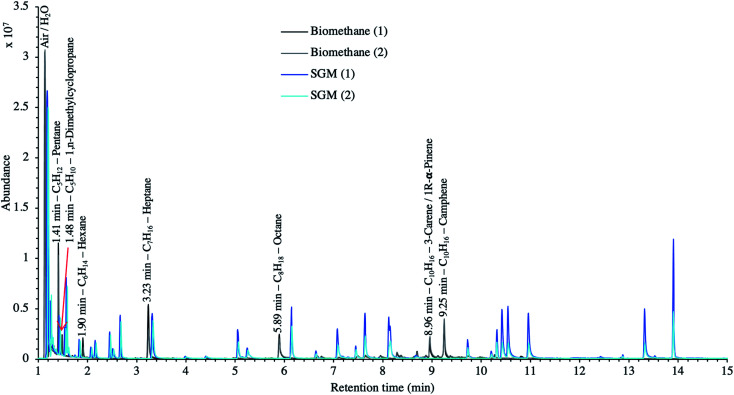
TIC of two biomethane replicates preconcentrated directly at 40 bar_a_ compared to the TIC of two 41 HVOC SGM replicates sampled and analyzed under the same conditions: 2 L_N_ collected at 40 bar_a_ on TA14-CpX29 MAT at 1 L_N_ min^−1^ in the HPTS.

Finally, to make up for the lacking TC quantification and merely as a semi-quantitative indication, Fig. 12 compares the TIC of a biomethane sample to the TIC of the 41 HVOC SGM sampled and analyzed under the same conditions (2 L_N_ collected at 40 bar_a_ on TA14-CpX29 MAT at 1 L_N_ min^−1^). The relatively high variability in signal intensities between replicates of a given sample in Fig. 12 is due to the poor nCx-TD prototype repeatability, as demonstrated earlier.^38^ Nonetheless, and disregarding differences in ionization efficiencies between TC present in the biomethane sample and in the SGM, the order of magnitude of the concentration threshold at which TC are present in the biomethane can be roughly estimated (50% error) from Fig. 12 inasmuch as all compounds in the SGM are certified to be present at 1 ppm_mol_. Most obvious TC in this biomethane sample (labelled on Fig. 12) hence seem to have a ≤1 ppm_mol_ concentration threshold considering the similarity of their peak signal intensities to the peaks of the SGM compounds. Other TC in the biomethane probably lurk at lower concentrations.

## 4. Conclusions and perspectives

A versatile field sampling method to easily preconcentrate trace compounds (TC) in gas samples at high working pressures (≤200 bar_a_) directly *in situ* on gas production sites has been presented. The high-pressure adsorbent tube sampling (HPTS) prototype hosting a TA14-CpX29 multibed adsorbent tube has been successfully applied to preconcentrate TC in a pressurized synthetic gas and a grid-injected biomethane. In the pressure range 5–100 bar_a_, handled in French gas transport grids, the gas sampling pressure had no effect on the preconcentration of TC on the adsorbent tubes when the gas circulates through the tube and as long as the adsorbents are not saturated. The TA14-CpX29 multibed adsorbent tubes were found appropriate to preconcentrate, in a single sampling run, a wide range of volatile organic TC families in the synthetic gas and the biomethane: halogenated compounds, (poly)cyclic- and linear alkanes, alkenes, terpenes, aromatic compounds, sulphur-compounds. Semi-quantification indicated pentane, dimethylcyclopropane, hexane, heptane, octane, α-pinene and camphene are present at a ≤1 ppm_mol_ concentration threshold in the biomethane.

Regarding real gas sampling for TC determination, combining an efficient preconcentration support such as multibed adsorbent tubes with the HPTS prototype enables to circumvent the disadvantages of whole gas sampling where transport and subsequent transfer to a preconcentration unit are required. With direct *in situ* high pressure preconcentration of TC in pressurized gases, pressure regulators are bypassed, shortening the sampling line upstream preconcentration, hence diminishing contamination risks and TC loss risks by sorption onto surfaces in surplus valves, connectors and tubing. The preconcentration unit (here a multibed adsorbent tube) is directly plugged into the gas pipeline, avoiding transfers from a whole gas sampling vessel and associated contamination and TC loss risks by sorption to transfer lines. Additionally, adsorbent tubes shipment to the lab is easy, fast and secure in view of their small sizes and of the absence of the flammable gas matrix (in the case of biomethane). As moreover TC stability on adsorbent tubes is higher than in whole gas sampling vessels,^21,27^ sample storage stability issues are avoided.

It is believed the novel instrumentation presented will substantially help improving field sampling campaigns for the characterization of trace compounds in pressurized gas samples such as biomethane.

## List of abbreviations

CpX: Carbopack™X
GC: Gas chromatography
HPTS: High-pressure tube sampling prototype
HVOC: Halogenated volatile organic compound
ID: Internal diameter
L: length
MAT: Multibed adsorbent tube
MS: Mass spectrometry
PTFE: Polytetrafluoroethylene
RA: Relative abundance
SGM: Synthetic gas mixture
TA: Tenax®TA
TC: Trace compound(s)
TD: Thermodesorption
TD-GC-MS: Thermodesorption – gas chromatography – mass spectrometry
THT: Tetrahydrothiophene
TIC: Total ion current chromatogram

## Conflicts of interest

The authors declare no competing financial interests or personal relationships that could have influenced the work reported in this paper.

## Supplementary Material

RA-012-D2RA00601D-s001
