# Supplementary material for: Novel field-portable high-pressure adsorbent tube sampler prototype for the direct in situ preconcentration of trace compounds in gases at their working pressures: application to biomethane
Source: RSC Adv. 2022 Mar 30;12(16):10071–87. doi: 10.1039/d2ra00601d (PMC8966642; doi:10.1039/d2ra00601d)
Supplement: RA-012-D2RA00601D-s001 [file RA-012-D2RA00601D-s001.pdf]

Electronic Supplementary Material (ESI) for RSC Advances.
This journal is © The Royal Society of Chemistry 2022

Supplemental Information

Novel field-portable high-pressure adsorbent tube sampler prototype for the direct *in situ* preconcentration of trace compounds in gases at their working pressures: application to biomethane

Aurore Lecharlier[1,2], Hervé Carrier[1], Brice Bouyssiere[2], Guilhem Caumette[3], Pierre Chiquet[3], Isabelle Le Hécho[2*]

[1] Universite de Pau et des Pays de l'Adour, E2S UPPA, CNRS, TOTAL, LFCR UMR 5150, BP 1155 avenue de l'Université, 64013 Pau Cedex, France
[2] Universite de Pau et des Pays de l'Adour, E2S UPPA, CNRS, IPREM UMR 5254, Technopôle Hélioparc, 2 avenue du Président Angot, 64053 Pau Cedex 09, France
[3] Terēga, 40 Avenue de l'Europe, CS 20 522, 64010 Pau Cedex, France

***Corresponding Author:** Isabelle Le Hécho • isabelle.lehecho@univ-pau.fr

Authors' ORCID n°:
Aurore Lecharlier https://orcid.org/0000-0002-0309-7464
Hervé Carrier https://orcid.org/0000-0002-1452-7933
Brice Bouyssiere https://orcid.org/0000-0001-5878-6067
Isabelle Le Hécho https://orcid.org/0000-0002-9751-7553

1
Supplemental Information

Theoretical note on multibed adsorbent tubes

Multibed adsorbent tubes (MAT) are attractive preconcentration supports for complex gas samples with unknown composition such as biomethane in view of the large diversity of chemical TC families it can contain [1–6] with associated large boiling points- and polarity-ranges [5,7]. While not any adsorbent is universal enough to adsorb all TC [5,8], the working principle of a MAT precisely enables to preconcentrate a large range of TC in a wide volatility range in one single sampling run. In a MAT, different adsorbents are arranged in order of increasing sorption strength (increasing surface area, decreasing pore size) in the gas sampling direction [9,10]. As the gas matrix (CH_4 in the case of biomethane) passes through the tube without being retained due to its too high volatility, the weak front adsorbent (here Tenax®TA) traps relatively large, heavy, high-boiling TC (boiling point > ~80°C) but is not strong enough to retain small volatile nor very volatile TC (boiling point < ~50 – 80°C). Those hence move onwards to the next gradually stronger adsorbent beds (here Carbopack™X) whereon they eventually get adsorbed. Importantly, thermodesorption of MAT must occur in the reverse direction as compared to the gas sampling direction. Thermodesorbing a MAT in the same direction as sampling would result in the carrier gas of the TD to blow high-boiling compounds desorbed from the weak front bed towards the stronger back bed whereon they could (partly) re-adsorb and not enter the GC-column. The critical benefits of MAT are that (1) the gas matrix is not retained enabling preconcentration ('isolation') of TC, (2) high-boiling TC never meet strong adsorbents whereon they would irreversibly adsorb, impeding their desorption upon analysis, and (3) very volatile TC can be trapped on and desorbed from strong adsorbents. Therefore, MAT enable quantitative adsorption and desorption (which is analytically at least as important as adsorption) of TC over a wider volatility and polarity range than single adsorbent beds do.

References

[1] F. Hilaire, E. Basset, R. Bayard, M. Gallardo, D. Thiebaut, J. Vial, Comprehensive Two-Dimensional Gas Chromatography for Biogas and Biomethane Analysis, Journal of Chromatography A. 1524 (2017) 222–232. https://doi.org/10.1016/j.chroma.2017.09.071.

[2] M. Ghidotti, D. Fabbri, C. Torri, Determination of Linear and Cyclic Volatile Methyl Siloxanes in Biogas and Biomethane by Solid-Phase Microextraction and Gas Chromatography-Mass Spectrometry, Talanta. 195 (2019) 258–264. https://doi.org/10.1016/j.talanta.2018.11.032.

[3] S. Rasi, J. Läntelä, J. Rintala, Trace Compounds Affecting Biogas Energy Utilisation – A Review, Energy Conversion and Management. 52 (2011) 3369–3375. https://doi.org/10.1016/j.enconman.2011.07.005.

[4] K. Arrhenius, A. Fischer, O. Büker, Methods for Sampling Biogas and Biomethane on Adsorbent Tubes After Collection in Gas Bags, Applied Sciences. 9 (2019) 1171. https://doi.org/10.3390/app9061171.
[5] K. Arrhenius, A.S. Brown, A.M.H. van der Veen, Suitability of Different Containers for the Sampling and Storage of Biogas and Biomethane for the Determination of the Trace-Level Impurities – A Review, Analytica Chimica Acta. 902 (2016) 22–32. https://doi.org/10.1016/j.aca.2015.10.039.
[6] K. Arrhenius, H. Yaghooby, L. Rosell, O. Büker, L. Culleton, S. Bartlett, A. Murugan, P. Brewer, J. Li, A.M.H. van der Veen, I. Krom, F. Lestremau, J. Beranek, Suitability of Vessels and Adsorbents for the Short-Term Storage of Biogas/Biomethane for the Determination of Impurities – Siloxanes, Sulfur Compounds, Halogenated Hydrocarbons, BTEX, Biomass and Bioenergy. 105 (2017) 127–135. https://doi.org/10.1016/j.biombioe.2017.06.025.
[7] E. Gallego, F.J. Roca, J.F. Perales, X. Guardino, E. Gadea, Development of a Method for Determination of VOCs (including Methylsiloxanes) in Biogas by TD-GC/MS Analysis Using Supel™ Inert Film Bags and Multisorbent Bed Tubes, International Journal of Environmental Analytical Chemistry. 95 (2015) 291–311. https://doi.org/10.1080/03067319.2015.1016012.
[8] J. Brown, B. Shirey, A Tool for Selecting an Adsorbent for Thermal Desorption Applications. Technical Report, (2001). https://www.sigmaaldrich.com/content/dam/sigma-aldrich/docs/Supelco/General_Information/t402025.pdf (accessed April 23, 2020).
[9] US EPA, Compendium of Methods for the Determination of Toxic Organic Compounds in Ambient Air. Second Edition. Compendium Method TO-17: Determination of Volatile Organic Compounds in Ambient Air Using Active Sampling Onto Sorbent Tubes (EPA/625/R-96/010b), (1999). https://www3.epa.gov/ttnamti1/files/ambient/airtox/to-17r.pdf (accessed April 23, 2020).
[10] M. Harper, Review. Sorbent Trapping of Volatile Organic Compounds from Air, Journal of Chromatography A. 885 (2000) 129–151. https://doi.org/10.1016/S0021-9673(00)00363-0.

2
Supplemental Tables

*Table SI-1: Chromatographic retention times (min) of compounds identified from the TD-GC-MS analysis of the SGM sampled at different test-pressures and different volumes at 1 $L_N \cdot min^{-1}$ on the TA14-CpX29 MAT in the HPTS prototype. STDEV : standard deviation. * : absent. ◊ : co-elution of tetrachloromethane, acrylonitrile and benzene*

**Sampling pressure (bar_a)**	**100**		**40**					**5**	
**Sampled volume (L_N)**	**2**		**2**		**5**	**1**		**2**	
**Replicates**	n=3		n=2		n=1	n=2		n=3	
**Compounds from the SGM**	**Mean**	**STDEV**	**Mean**	**STDEV**	**Value**	**Mean**	**STDEV**	**Mean**	**STDEV**
Dichlorodifluoromethane	**1.217**	0.018	**1.227**	0.018	**1.217**	**1.250**	0.006	**1.245**	0.030
Chloromethane	*	*	*	*	*	*	*	*	*
Chloroethene	**1.270**	0.013	**1.282**	0.025	**1.267**	**1.300**	0.005	**1.294**	0.030
1,3-Butadiene	**1.275**	0.023	**1.283**	0.008	**1.28**	**1.311**	0.006	**1.306**	0.030
1,2-Dichloro-1,1,2,2-tetrafluoroethane	**1.244**	0.017	**1.254**	0.018	**1.245**	**1.277**	0.006	**1.271**	0.031
Bromomethane	**1.325**	0.017	**1.334**	0.017	**1.326**	**1.357**	0.006	**1.352**	0.030
Chloroethane	**1.345**	0.017	**1.354**	0.017	**1.348**	**1.377**	0.006	**1.371**	0.029
Trichloromonofluoromethane	**1.427**	0.016	**1.436**	0.017	**1.429**	**1.459**	0.006	**1.453**	0.029
1,1-Dichloroethene	**1.538**	0.015	**1.546**	0.016	**1.54**	**1.568**	0.006	**1.563**	0.028
Dichloromethane	**1.602**	0.015	**1.611**	0.016	**1.606**	**1.633**	0.006	**1.627**	0.027
1,1,2-Trichloro-1,2,2-trifluoroethane	**1.569**	0.015	**1.577**	0.016	**1.571**	**1.599**	0.006	**1.594**	0.028
1,1-Dichloroethane	**1.833**	0.014	**1.841**	0.016	**1.836**	**1.863**	0.006	**1.857**	0.027
cis-1,2-Dichloroethene	**2.070**	0.014	**2.079**	0.015	**2.073**	**2.101**	0.006	**2.093**	0.025
Trichloromethane	**2.157**	0.014	**2.164**	0.016	**2.159**	**2.185**	0.007	**2.178**	0.025
1,1,1-Trichlorethane	**2.451**	0.014	**2.457**	0.014	**2.452**	**2.479**	0.006	**2.471**	0.025
Tetrachloromethane ◊	**2.664**	0.013	**2.671**	0.013	**2.666**	**2.691**	0.007	**2.684**	0.025
Acrylonitrile ◊	**2.664**	0.013	**2.671**	0.013	**2.666**	**2.691**	0.007	**2.684**	0.025
Benzene ◊	**2.664**	0.013	**2.671**	0.013	**2.666**	**2.691**	0.007	**2.684**	0.025
1,2-Dichlorethane	**2.517**	0.015	**2.525**	0.015	**2.516**	**2.548**	0.007	**2.537**	0.024
Trichloroethene	**3.317**	0.013	**3.323**	0.014	**3.318**	**3.345**	0.008	**3.335**	0.024
1,2-Dichloropropane	*	*	*	*	*	*	*	*	*
cis-1,3-Dichloropropene	**4.401**	0.015	**4.413**	0.014	**4.389**	**4.447**	0.001	**4.416**	0.017
Toluene	**5.056**	0.011	**5.060**	0.011	**5.058**	**5.077**	0.006	**5.068**	0.018
trans-1,3-Dichloropropene	**5.139**	0.017	**5.159**	0.022	**5.119**	**5.270**	0.004	**5.149**	0.014
1,1,2-Trichlorethane	**5.242**	0.011	**5.251**	0.011	**5.233**	**5.201**	/	**5.250**	0.014
Tetrachloroethene	**6.145**	0.008	**6.148**	0.007	**6.15**	**6.159**	0.004	**6.153**	0.011
Chlorobenzene	**7.080**	0.009	**7.083**	0.008	**7.077**	**7.098**	0.001	**7.085**	0.010
1,2-Dibromoethane	**6.103**	0.015	**6.119**	0.007	**6.087**	**6.150**	0.008	**6.114**	0.009
Ethylbenzene	**7.452**	0.004	**7.456**	0.004	**7.452**	**7.463**	0.001	**7.458**	0.004
p-Xylene	**7.637**	0.006	**7.639**	0.005	**7.649**	**7.648**	0.002	**7.642**	0.007
m-Xylene	**7.637**	0.006	**7.639**	0.005	**7.649**	**7.648**	0.002	**7.642**	0.007
o-Xylene	**8.149**	0.010	**8.153**	0.005	**8.163**	**8.161**	0.001	**8.154**	0.010
Styrene	**8.127**	0.010	**8.128**	0.008	**8.13**	**8.139**	0.000	**8.130**	0.009
1,1,2,2-Tetrachlorethane	**8.663**	0.013	**8.676**	0.011	**8.638**	**8.694**	0.003	**8.661**	0.007
1,3,5-Trimethylbenzene	**9.724**	0.002	**9.727**	0.002	**9.727**	**9.730**	0.001	**9.727**	0.002
1,2,4-Trimethylbenzene	**10.200**	0.003	**10.204**	0.003	**10.2**	**10.207**	0.000	**10.202**	0.001
1,3-Dichlorobenzene	**10.423**	0.006	**10.425**	0.006	**10.422**	**10.436**	0.000	**10.424**	0.005
1,4-Dichlorobenzene	**10.546**	0.005	**10.548**	0.006	**10.547**	**10.556**	0.000	**10.548**	0.004
1,2-Dichlorobenzene	**10.960**	0.006	**10.963**	0.007	**10.959**	**10.974**	0.001	**10.962**	0.006
1,2,4-Trichlorobenzene	**13.323**	0.005	**13.324**	0.004	**13.324**	**13.333**	0.001	**13.324**	0.003
Hexachloro-1,3-Butadiene	**13.903**	0.001	**13.904**	0.001	**13.913**	**13.904**	0.001	**13.906**	0.002

3
Supplemental Figures

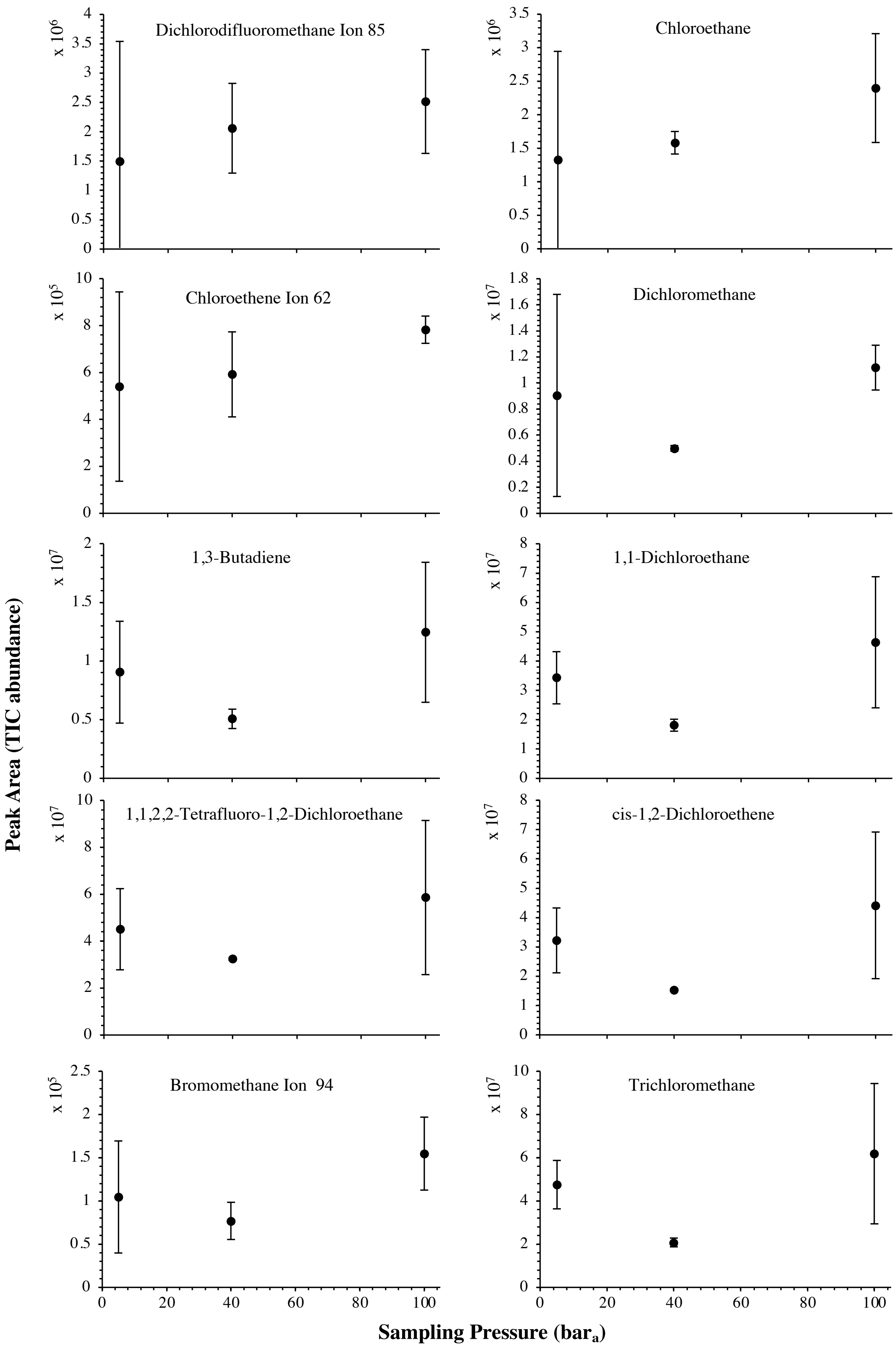

Figure SI-1: High-pressure adsorption isotherms of the HVOC not shown in Fig.4 of the core paper for test-condition A (2 L_N of the SGM sampled at 5, 40 and 100 bar$_a$ on TA14-CpX29 MAT). Average peak area with indication of the standard deviation.

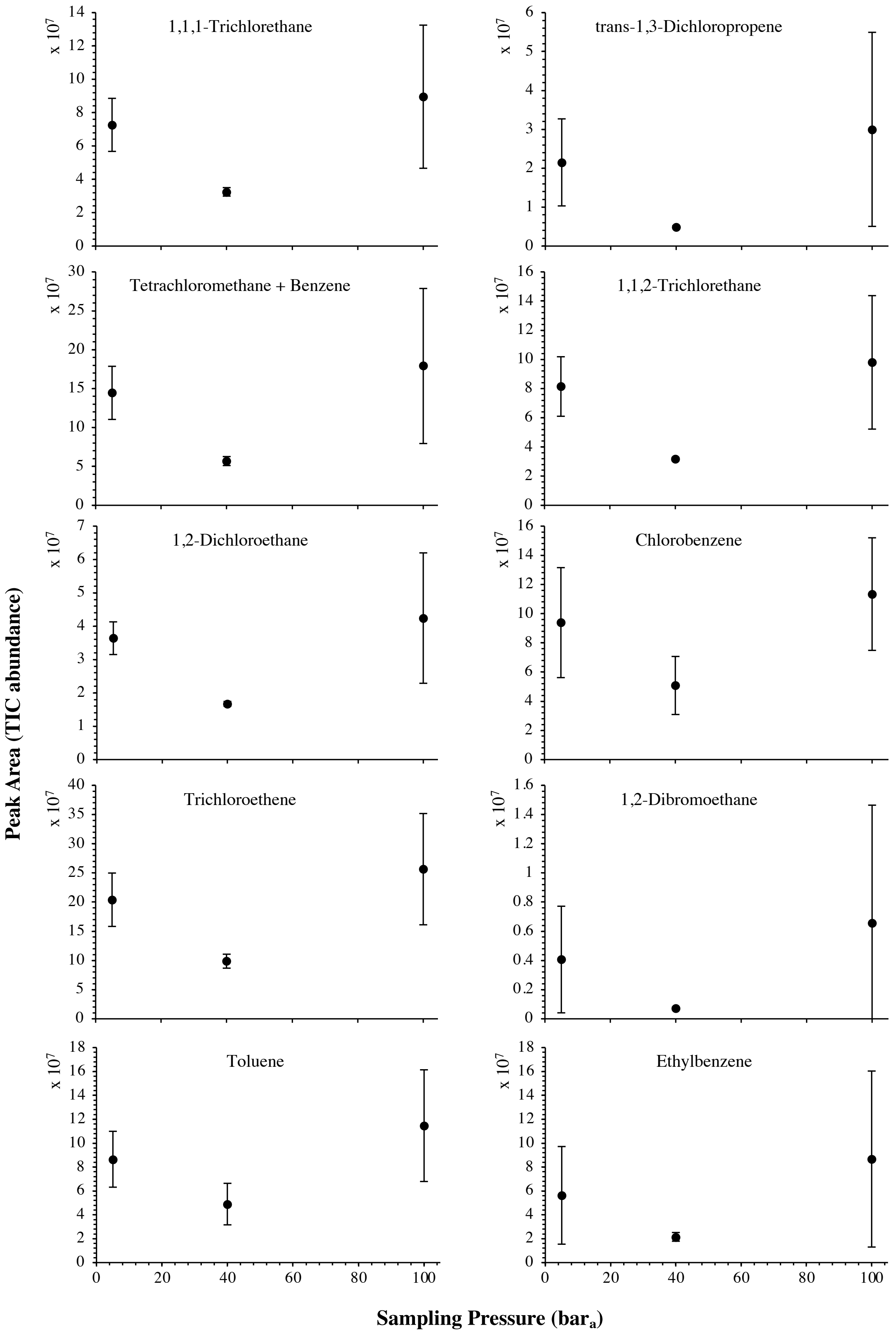

Figure SI-1: continued (1).

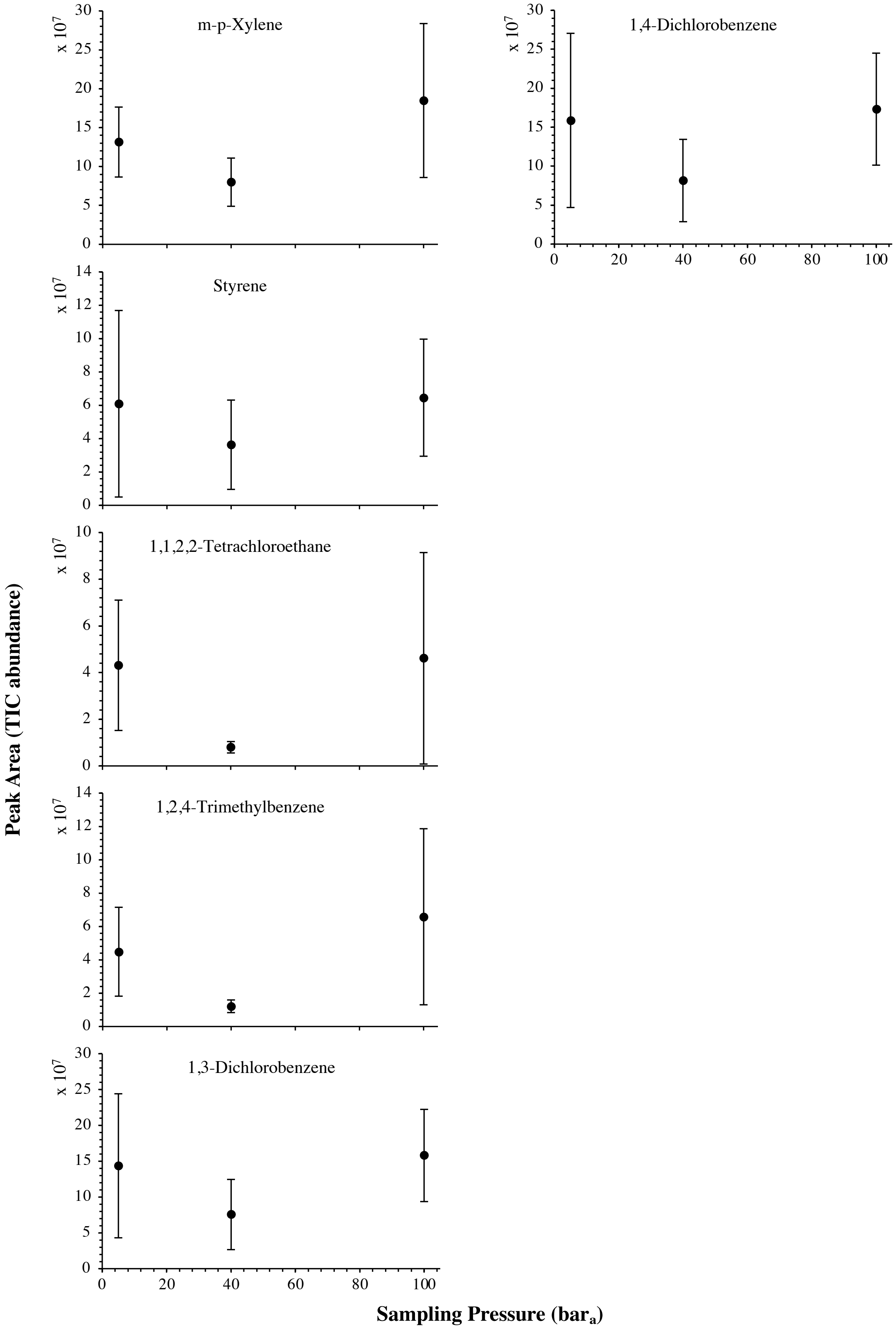

Figure SI-1: continued (2).

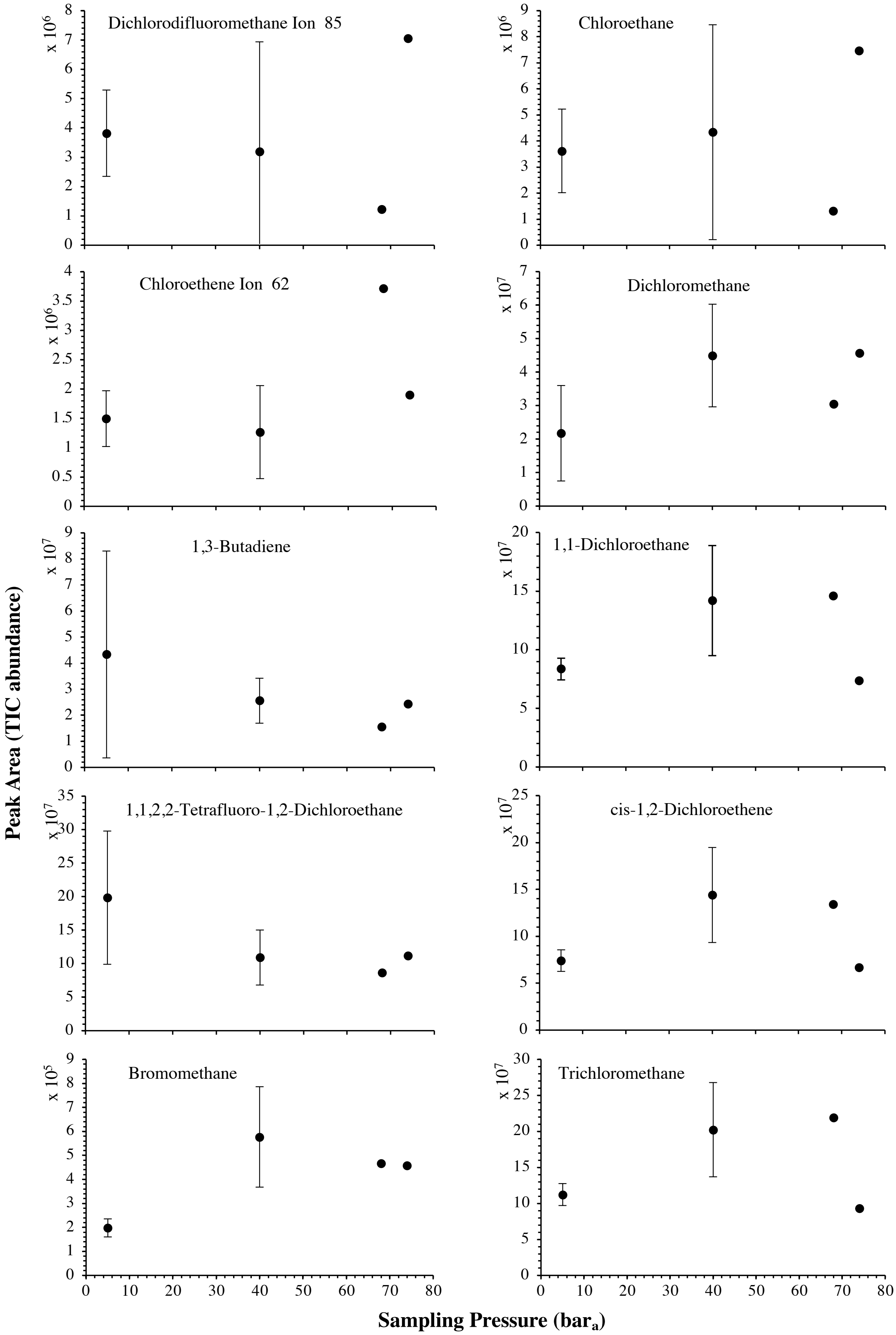

Figure SI-2: High-pressure adsorption isotherms of the HVOC not shown in Fig.5 of the core paper for test-condition B (5 L_N of the SGM sampled at 5, 40, 68 and 74 bar_a on TA14-CpX29 MAT). Average peak area with indication of the standard deviation.

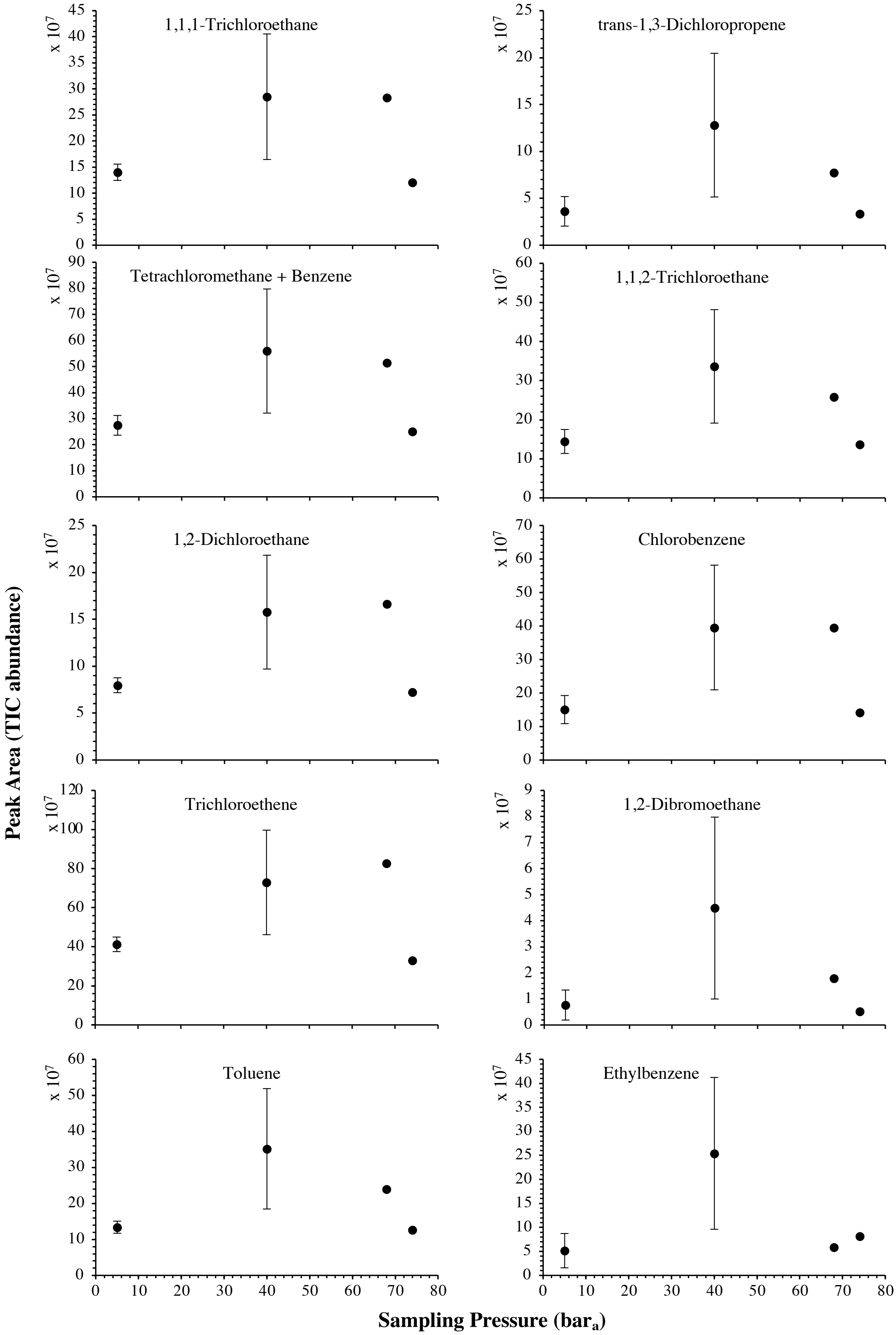

Figure SI-2: continued (1).

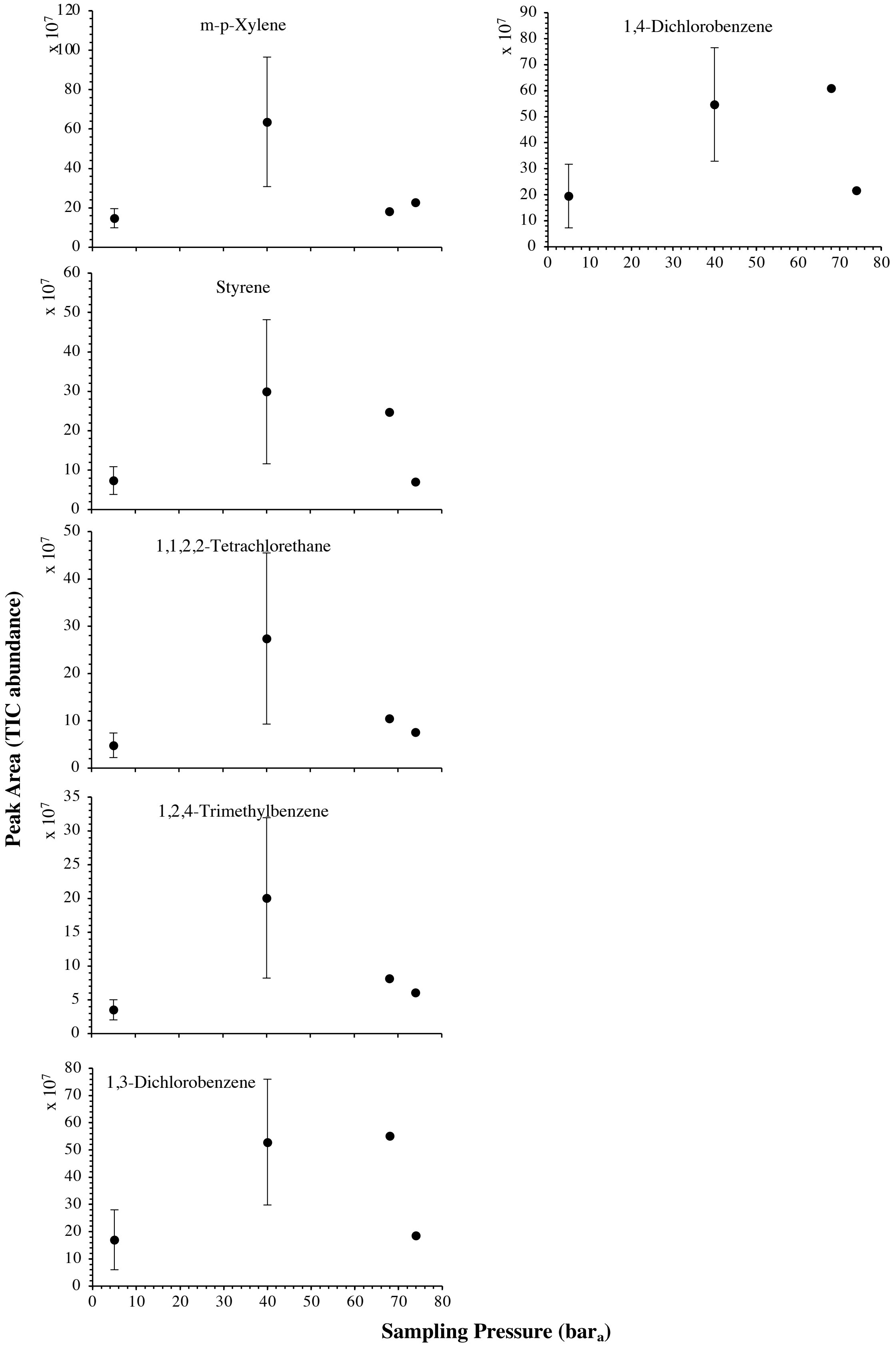

Figure SI-2: continued (2).

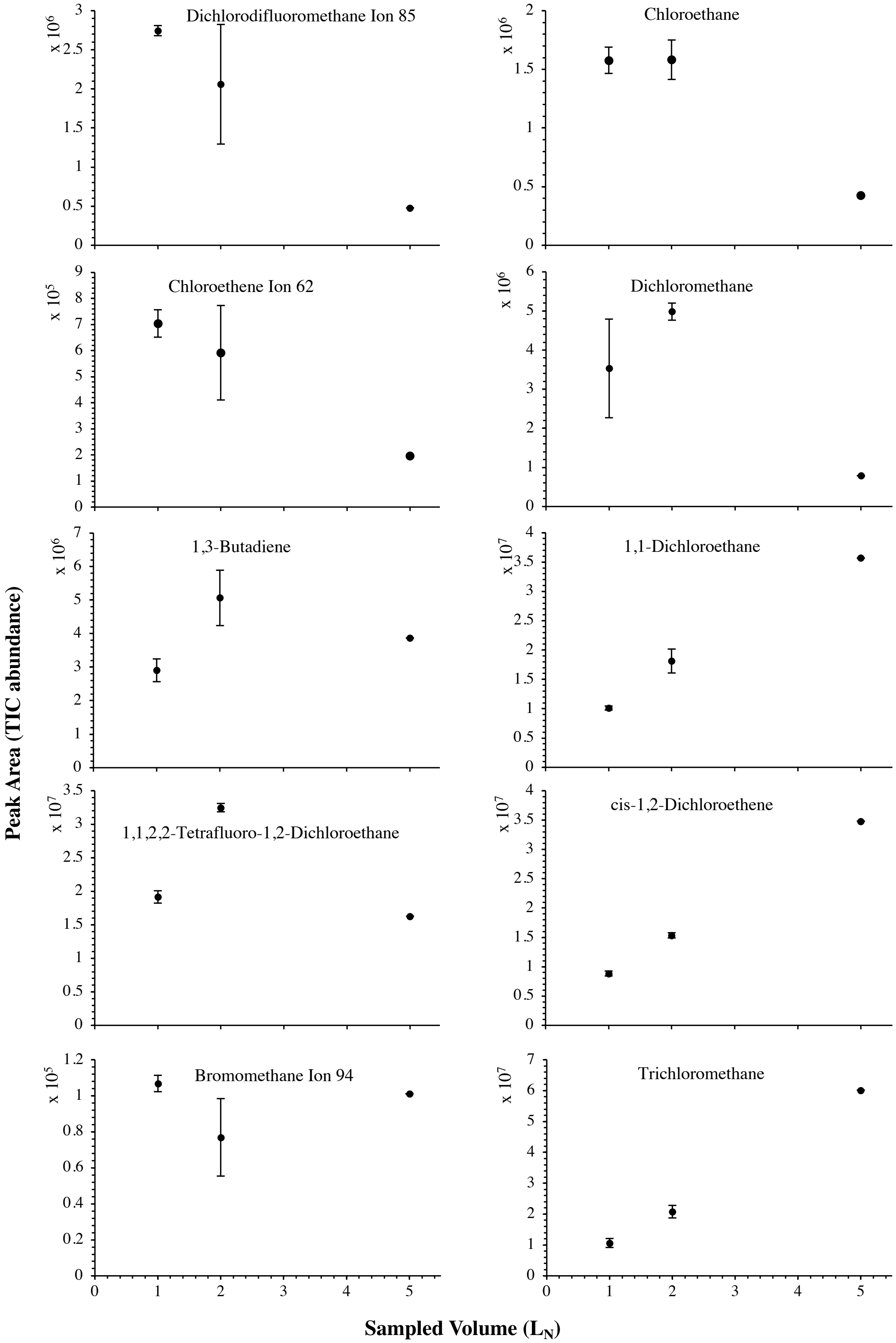

Figure SI-3: Partial breakthrough curves for the HVOC not shown in Fig.6 of the core paper for test-condition C (1, 2 and 5 L_N of the SGM sampled at 40 bar_a on TA14-CpX29 MAT). Average peak area with indication of the standard deviation.

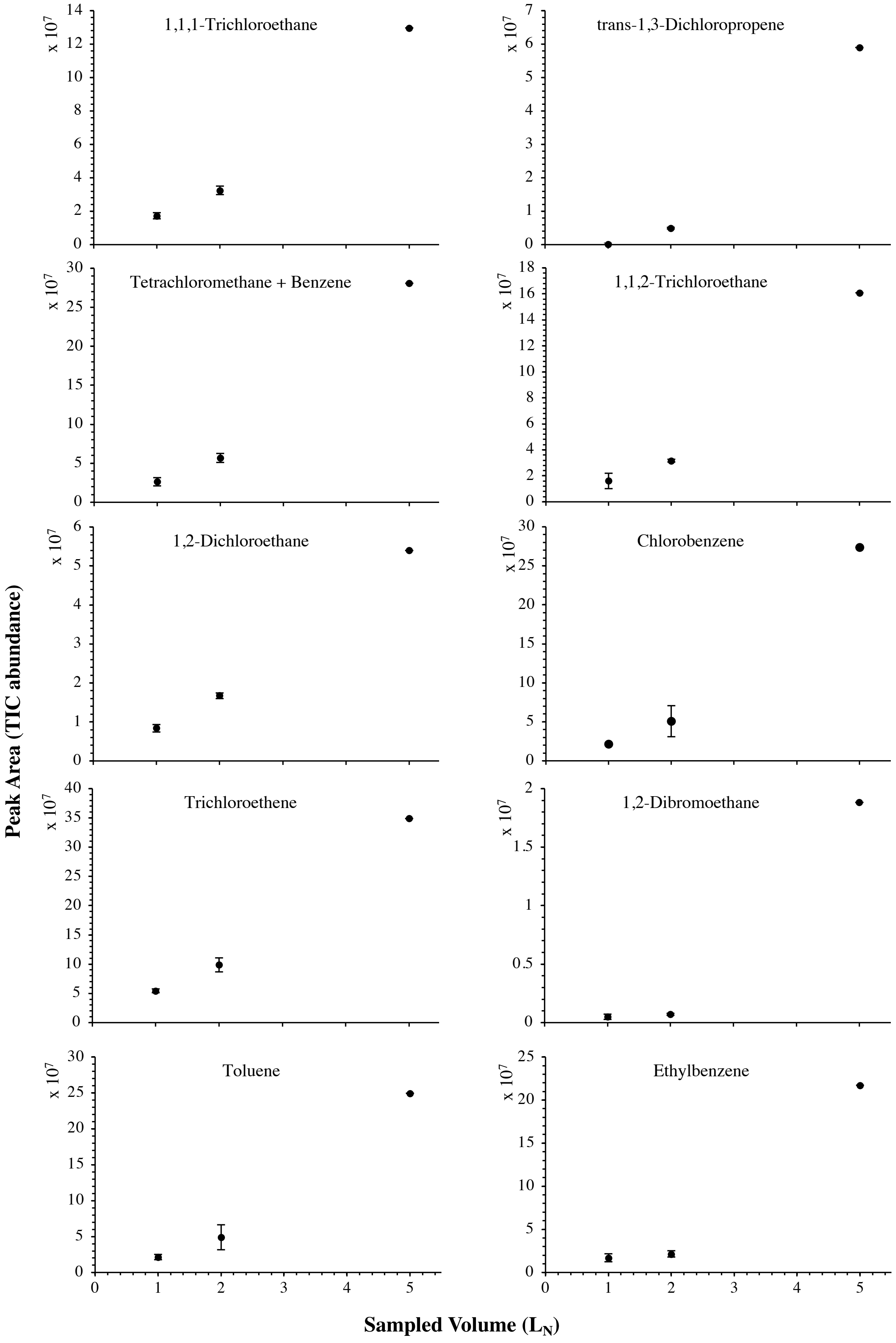

Figure SI-3: continued (1).

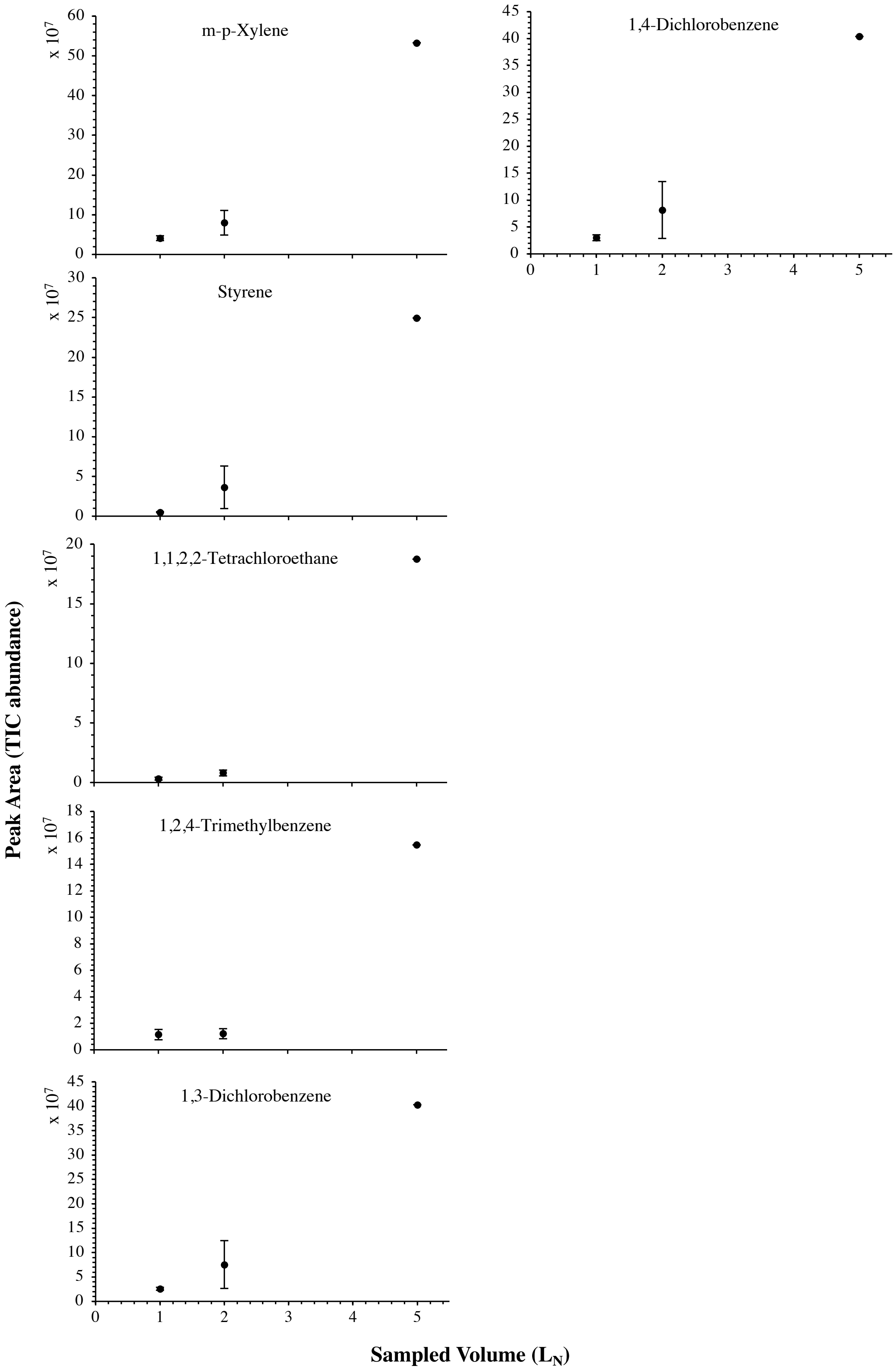

Figure SI-3: continued (2).

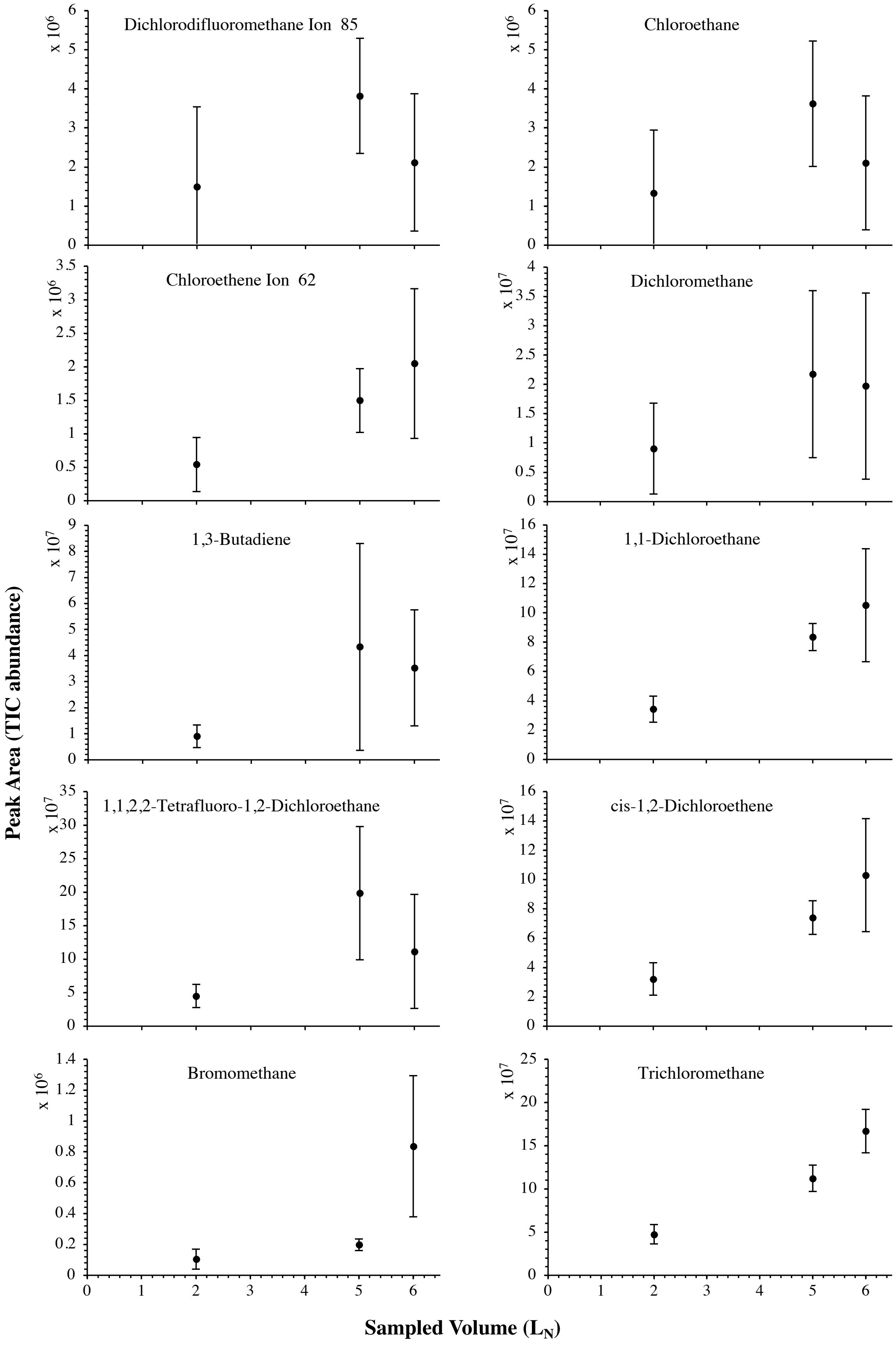

Figure SI-4: Partial breakthrough curves for the HVOC not shown in Fig.7 of the core paper for test-condition D (2, 5 and 6 L_N of the SGM sampled at 5 bar_a on TA14-CpX29 MAT). Average peak area with indication of the standard deviation.

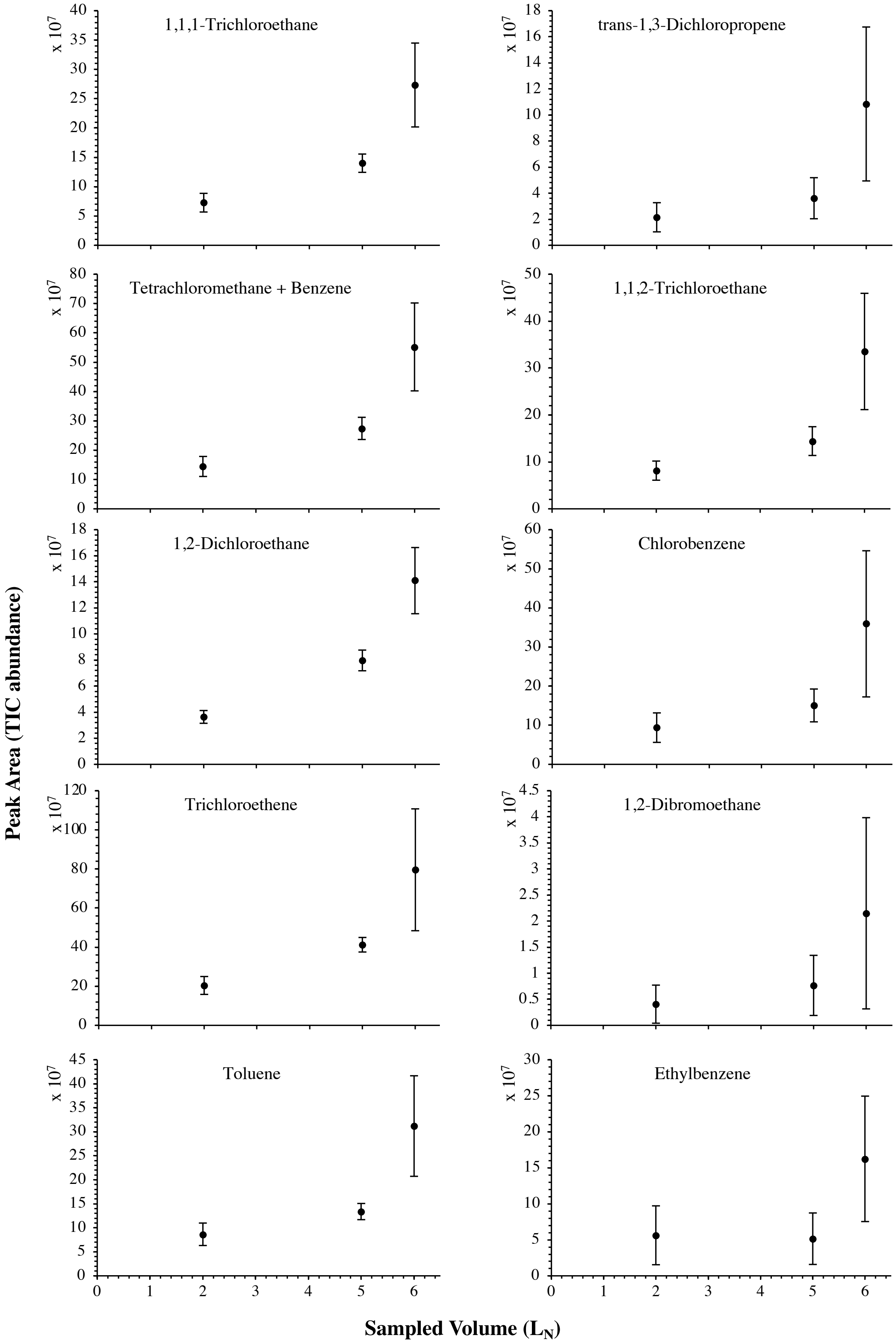

Figure SI-4: continued (1).

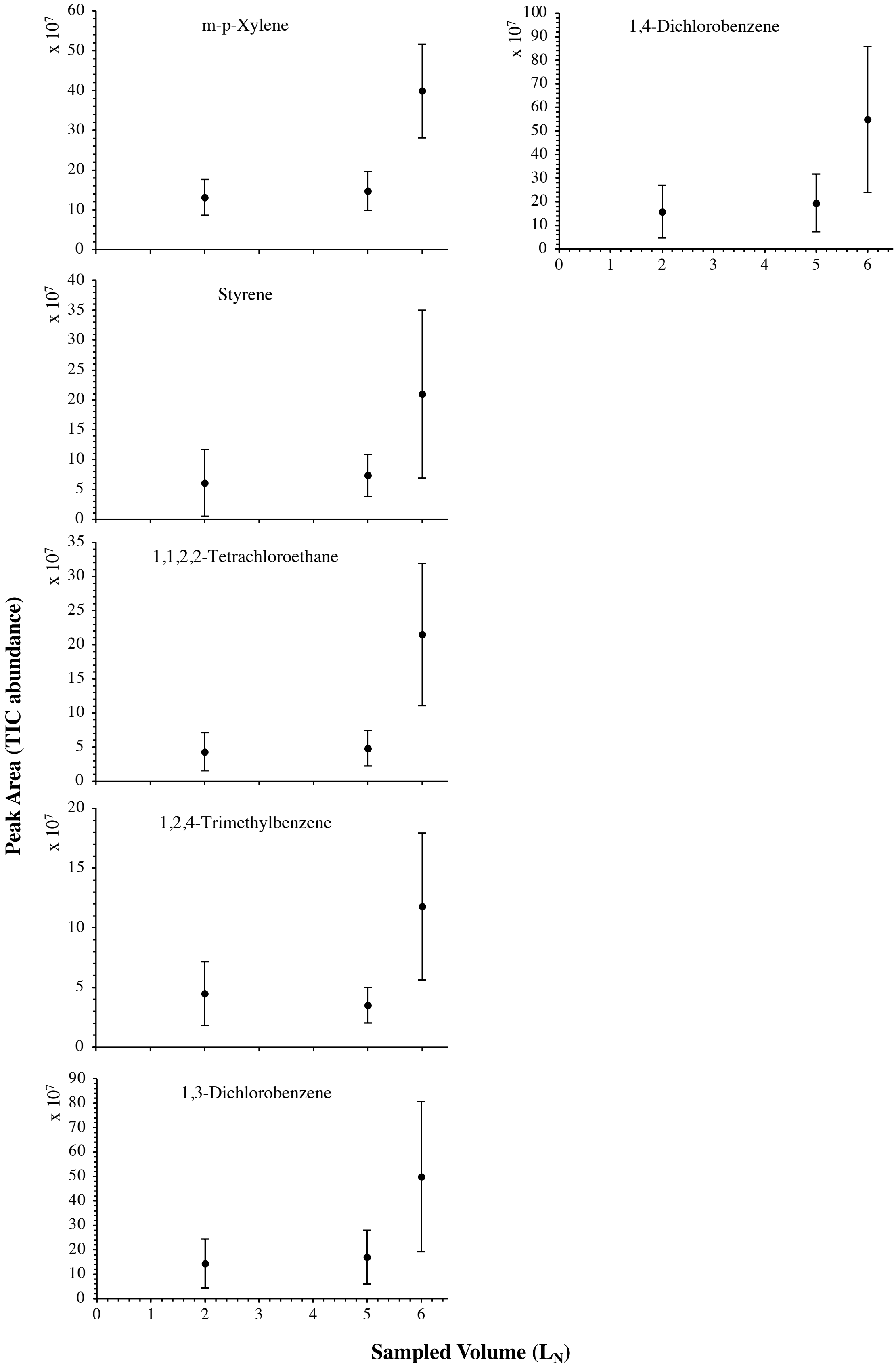

Figure SI-4: continued (2).
